# The LiPP Benchmark Set for Modeling Lipid–Protein Complexes: Comparison of Co-Folding and Docking Methods

**DOI:** 10.1021/acs.jcim.6c01457

**Published:** 2026-06-10

**Authors:** Li-Yen Yang, Shreyas Gupta, Lauren N. Mullininx, Andrew C. McShan

**Affiliations:** † School of Chemistry and Biochemistry, 1372 Georgia Institute of Technology, Atlanta, Georgia 30332, United States; ‡ School of Biological Sciences, Georgia Institute of Technology, Atlanta, Georgia 30332, United States

## Abstract

Lipid–protein interactions are a universal feature of nearly all cellular pathways and are essential for advances in medicine, pharmaceuticals, and biotechnology. However, computational prediction of lipid–protein binding poses lags behind protein–protein, protein–small molecule, and protein–nucleic acid complexes, partially due to the lack of inclusive data sets of ground truth structures. Here, we introduce the Lipid–Protein Poses (LiPP) benchmark, a curated data set of 311 nonredundant nonannular lipid–protein complex structures that sample a range of protein folds and lipid types. Using LiPP, we systematically evaluate the performance of three AI-based cofolding tools (AlphaFold 3, Chai-1, RoseTTAFold AA), one AI-based docking tool (DiffDock-L), and one physics-based docking tool (AutoDock Vina) to model lipid–protein complexes. Lipid binding poses generated by different software vary in physical plausibility: physics-based docking (AutoDock Vina) and some AI predictors (AlphaFold 3, Chai-1) largely preserve intramolecular and intermolecular constraints, whereas other AI methods (DiffDock-L, RoseTTAFold AA) frequently violate these constraints. AlphaFold 3 demonstrates the highest success rate at 76.1% under the RMSD < 2 Å criterion; however, the success rate drops to 47.2% for structures not seen during training. We identify notable differences in the ability of each software to generate reliable confidence metrics for discriminating accurate from inaccurate lipid binding poses. Our results underscore a substantial need to improve both AI- and physics-based methods for modeling lipid–protein interactions where considerations in lipid size, class, and flexibility are important. The LiPP benchmark set provides a new standardized platform to probe a range of lipid–protein complex modeling tasks. LiPP highlights intrinsic shortcomings in modern docking and cofolding approaches for capturing lipid–protein interaction features and guides the development of next-generation computational tools for advancing lipid biology research.

## Graphical abstract

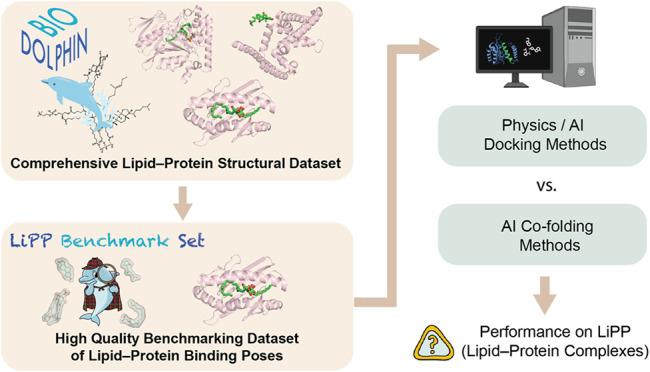

## Introduction

Lipids are a chemically diverse group of hydrophobic or amphipathic biomolecules that are largely insoluble in water but soluble in organic solvents, membranes, or when bound to proteins. Lipid–protein interactions are fundamental to biology where they orchestrate processes from membrane structure and energy metabolism to signaling, immune regulation, enzyme cofactors, pigments, and other key cellular functions.^1–4^ Lipid–protein interactions are also emerging as important tools for biotechnology and medicine, highlighted by lipid nanoparticle-based drug delivery, lipid-based vaccine adjuvants, and therapies to modulate lipid–protein interactions.^5–7^ LIPID MAPS classifies lipids into eight broad categories: 1. fatty acyls, 2. glycerolipids, 3. glycerophospholipids, 4. sphingolipids, 5. sterols, 6. prenols, 7. saccharolipids, and 8. polyketides^8–10^ (Figure 1). The eight classes of lipids differ dramatically in functional group chemistry, size, polarity, geometry, and flexibility. Consistent with this chemical and structural diversity, lipid–protein interactions also span a vast and heterogeneous landscape, ranging from nonspecific contacts stabilizing membrane-embedded proteins to highly specific contacts within buried pockets of soluble proteins.^11^ The remarkable heterogeneity of lipid–protein interactions presents unique challenges for computational modeling, motivating the need for curated benchmark data sets that sample representative examples across the eight major lipid classes.^12^

**Figure 1:**
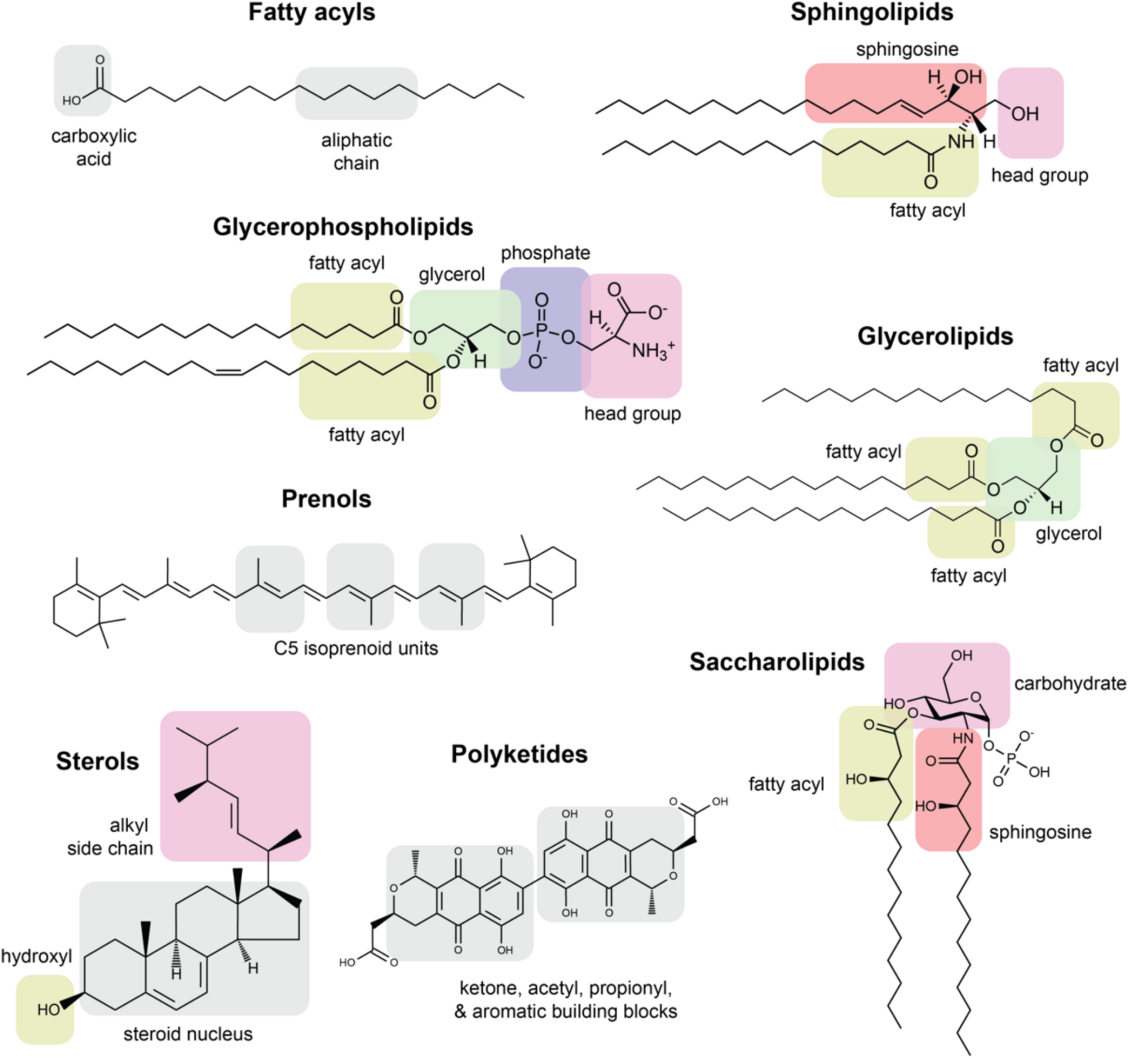
Representative structures of the eight classes of lipids as defined by LIPID MAPS. Example chemical structures for each of the eight lipid classes as defined by LIPID MAPS. PubChem Compound IDs (CID) are provided for each example. Fatty acyls: stearic acid, PubChem CID 5281; Sphingolipids: C15 ceramide, PubChem CID 86269023; Glycerophospholipids: 16:0–18:1 PS, PubChem CID 46891789; Glycerolipid: tripalmitin, PubChem CID 11147; Prenols: β-carotene, PubChem CID 5280489; Sterols: ergosterol, PubChem CID 444679; Polyketides: actinorhodin, PubChem CID 381343764; Saccharolipids: lipid X, PubChem CID 123907. The colored boxes highlight representative examples of specialized chemical units and functional groups relevant to each lipid class.

Lipid–protein interactions of membrane-embedded proteins, peripheral membrane proteins, and soluble proteins can be grouped into three regimes: bulk interactions, surface (annular) interactions, and buried (nonannular) interactions.^13–16^ Bulk lipids are not directly in contact with proteins. Nevertheless, bulk lipids are not passive components and can influence protein structure and function by modulating membrane properties, such as fluidity, diffusion, and curvature.^17^ Surface (annular) contacts involve lipids that occupy the immediate vicinity of a protein but would not be considered biological ligands of interest. Annular lipid–protein interactions are direct but largely nonspecific whereby the hydrophobic acyl chains of lipids form a dynamic, solvent-like shell around transmembrane regions of membrane proteins or hydrophobic surfaces of soluble proteins (Supporting Figure 1).^4^ For example, G protein–coupled receptors are membrane-embedded proteins surrounded by a dynamic shell of annular lipids, primarily glycerophospholipids and cholesterol, that modulate conformational landscape, ligand binding, and signaling through largely nonspecific interfacial interactions.^18^ A second example is membrane-embedded ABC transporters, where annular lipids surrounding the transmembrane domains influence conformational transitions associated with the transport cycle through largely nonspecific interfacial interactions.^19^ Annular lipid–protein interactions can be studied experimentally by embedding proteins into synthetic membrane mimetics for structural and biophysical studies, or through molecular dynamics simulations.^4,20,21^ Previous work has provided extensive insight into annular lipid–protein interactions; however, we do not focus here on these nonspecific interactions, despite their importance in lipid biology, membrane protein stability, and lipid-based biotechnology.^4,13,16,17,22^

Here, we focus on nonannular lipid–protein associations with ligand of relevance to the protein’s native biological function. Nonannular interactions form stable complexes within well-defined buried or partially buried protein pockets (Supporting Figure 1). Nonannular interactions confer molecular selectivity and are key for biochemical function.^23,24^ Defining features of nonannular lipid–protein complexes include shape complementarity, lipid headgroup-specific contacts, and electrostatic complementarity.^4,16,17^ In stark contrast to annular lipids, nonannular lipid–protein complexes are also characterized by nanomolar to micromolar affinities and slower exchange kinetics.^25–30^ The assembly of these lipid–protein complexes can be due to intrinsic ability of proteins to extract lipids from membranes or through coordination with lipid transfer proteins.^15,31,32^ While nonannular interactions can occur for many classes of proteins, the regime is exemplified by soluble lipid-binding proteins and extracellular domains of integral membrane proteins (Supporting Figure 1). As a first example, nontransmembrane, soluble lipid transfer proteins sequester certain types of lipid molecules within a β-barrel fold that creates buried hydrophobic cavity.^31,33^ The lipid transfer protein LCN1, a major lipid-binding protein in tears, bind to sphingomyelin and phosphatidylcholine via cation−π interactions between aromatic residues with lipid choline headgroups.^32^ Notably, it does not bind structurally similar lipids lacking cationic choline headgroups.^32^ This selectivity enables lipid sequestration and contributes to lipid regulation within the tears.^34^ A different lipid transfer protein family, cellular retinol-binding proteins, binds all-*trans* retinol with high affinity within a hydrophobic β-barrel cavity, forming specific hydrogen-bonding and hydrophobic interactions.^35^ This selectivity enables controlled intracellular trafficking of retinol for metabolism and storage, thereby regulating retinoid homeostasis and downstream signaling pathways.^36^ A final example is the extracellular domain of the lipid antigen-presenting immunoreceptor CD1d, which employs a concerted network of hydrophilic and hydrophobic residues lining a mixed α/β fold to form a geometric selectivity filter for both lipid headgroups and acyl chains.^37,38^ CD1’s lipid antigen selectivity is critical for its role in presenting lipid antigens to T cell receptors.^39^ The structural, biological, functional, kinetic, and thermodynamic relevance of nonannular lipid–protein interactions is supported by NMR spectroscopy, cryo-EM and X-ray structures, and biophysical measurements across a wide range of systems.^31,37,40–43^ The ability to robustly and accurately model nonannular lipid–protein interactions is crucial for predicting lipid-binding modes, lipid specificity, the functional consequences of mutations, and biochemical outcomes.^21,44–46^

Approaches for modeling biomolecules and their complexes generally fall into three conceptual paradigms that differ in underlying assumptions and required inputs: molecular docking, structure prediction, and cofolding.^47,48^ Classical molecular docking methods predict the binding pose of a ligand within a protein pocket using predefined atomic coordinates provided by the user.^49^ Protein and ligand atoms can be treated as flexible, but the input coordinates are generally assumed to be close to the final bound conformation.^50^ Molecular docking tools, such as AutoDock Vina,^51^ GLIDE,^52^ and GOLD,^53^ predict small molecule–protein complex structures by combining physics-based scoring with heuristic or exhaustive sampling to explore possible ligand binding poses.^54^ The most plausible native-like binding pose is distinguished from less favorable poses using scoring functions that approximate binding energy. The balance between speed and computational efficiency for physics-based docking methods, dictated by the search algorithm, scoring function, and degree of flexibility allowed, imposes limitations on the accuracy and throughput of the methods.^55,56^ Alternative molecular docking approaches using deep learning have the potential to improve speed and accuracy.^57–60^ For example, deep learning-based methods centered on geometric graph neural networks (GNN) have made advancements in capturing spatial relationships between protein and ligand atoms, as exemplified by EquiBind^61^ and TankBind.^62^ In addition, DiffDock and DiffDock-L introduced generative diffusion models with an SE(3)-equivariant GNN to iteratively refine randomly sampled ligand poses over translation, rotation, and torsion angles through a denoising process.^63,64^

Beyond molecular docking, structure prediction methods predict atomic coordinates of proteins without considering bound ligands. Examples of structure prediction software include Rosetta,^65^ which predicts protein structures by generating conformational ensembles and selecting models based on a physics- and knowledge-based energy function, and AlphaFold 2,^66,67^ which predicts the structure of proteins directly from their amino acid sequences using coevolutionary information extracted from multiple sequence alignments. Relatedly, cofolding approaches, such as AlphaFold 3,^68^ Chai-1,^69^ and RoseTTAFold AA,^70^ predict structures of biomolecular complexes directly from user-provided sequences (i.e., amino acid sequence for proteins and SMILES string for ligands). In cofolding approaches, the ligand binding pocket and binding pose are typically not assumed to be predetermined; rather, they emerge through the process of jointly inferring protein conformation and ligand pose, enabling mutual structural adaptation during complex formation.^71^ Because molecular docking and cofolding approaches differ fundamentally in their inputs, assumptions, and architecture, they represent distinct modeling regimes rather than directly equivalent methods. There is ongoing debate regarding which approach performs better; cases in which physics-based methods prevail,^72^ as well as cases in which AI-based methods prevail,^73,74^ have both been reported, and results likely vary on a case-by-case basis depending on modeling parameters, software used, benchmark data set used, and other methodological factors.

For decades, the majority of research in the field of biomolecular modeling has focused on protein–protein, protein–small molecule, and protein–nucleic acid interactions.^55,75–80^ Beyond the inherent complexity of modeling lipid–protein complexes, there is also a lack of well-curated data sets of ground truth atomic structures to evaluate different modeling approaches and software. While benchmark data sets are readily available for small molecule–protein interactions (i.e., Astex Diverse,^81^ DockGen-E,^74^ and the PoseBusters Benchmark^72^), an analogous benchmark data set for lipid–protein interactions is currently lacking. Moreover, common data sets for training or calibrating molecular docking or cofolding algorithms, such as PDBbind,^82^ are also restricted to small drug-like molecules. Thus, a curated lipid–protein data set could help with fine-tuning the force fields of docking tools and the weights of neural networks in cofolding tools. Given the remarkable structural diversity and chemically distinct features of lipid molecules, the applicability of small molecule-tuned algorithms on lipid–protein interactions remains an open question. While an appreciable effort in the field has worked toward modeling lipid–protein complexes,^83–86^ the performance of modern modeling software across a wide range lipid–protein assemblies has not been systematically evaluated. Consequently, it remains unclear whether existing molecular docking and cofolding tools can reliably model the lipid–protein interactome.

In this study, we present the Lipid–Protein Poses (LiPP) benchmark set, a curated data set of 311 unique lipid–protein complex structures that partially captures the broad diversity in both protein folds and lipid classes. LiPP prioritizes high structural quality and data consistency rather than full coverage of the lipid–protein interactome, but nevertheless represents a valuable resource for benchmarking docking and cofolding tools against a range of lipid–protein modeling tasks. Researchers should therefore interpret the benchmark results presented here in the context of this data set curation principle. Collectively, our results highlight that both AI- and physics-based approaches require substantial improvements to address the complexities inherent to lipid–protein interactions, including conformational flexibility, diversity in physicochemical properties at binding interfaces, and molecular specificity. By providing a unified and well-curated benchmark, LiPP offers a valuable foundation for the future development of computational methods in lipid biology.

## Materials and Methods

### Curation of the LiPP Benchmark Set

We curated a data set of experimental structures of lipid–protein complexes to enable benchmarks on the accuracy of molecular docking and cofolding tools. The data set was collected from BioDolphin version 1.1,^12^ a comprehensive database for lipid–protein complex structures. We retrieved additional information, such as atom distance, ligand reports, structure release dates, and protein functional classifications of lipid–protein complexes, using the Protein Data Bank (PDB), RDKit, Biopython, and the PyMOL API.^87–91^ We followed similar procedures previously described to generate the PoseBusters Benchmark set (small molecule–protein complexes) to filter and retain only high-quality experimental data.^72^ From a total of 127,359 PDB entries in BioDolphin, each containing a single lipid–protein pair, we selected X-ray structures with resolutions better than 2 Å. Then, complexes containing unknown atom types or nonstandard amino acid residues were eliminated. We also excluded the PDB entries if they contained lipids failing the RDKit sanitization test, were incomplete, or formed covalent bonds with the proteins. PDB ligand reports were used to remove complexes containing lipids with stereochemical errors or atomic clashes. We then filtered out complex structures if the minimum distance between the lipid and protein is greater than 5 Å or less than 0.2 Å. We removed PDB entries in which the lipids were within 5 Å of any protein symmetry mate in the biological assemblies of the original PDB structure. To identify redundant lipid–protein pairs, we clustered the proteins by sequence similarity using the DIAMOND clustering algorithm^92^ (diamond cluster command) using an identity threshold of 90%. Within each protein cluster, if multiple proteins were complexed with the same lipid (identical Chemical Component Dictionary (CCD) ID code), we retained a single randomly chosen lipid–protein pair to avoid over-representation. Finally, we manually curated entries by visually inspecting PDB structures and evaluating the associated manuscripts to identify protein function and the relevance of the bound lipid. The nonredundant nonannular LiPP benchmark set for downstream analyses contains 331 lipid–protein pairs.

### Curation of the LiPP Test Set, Precutoff Set, and Similarity Assessments between the Different LiPP Data Sets

October first 2021 represents the date after the most recent training/calibration cutoff date for the five docking and cofolding methods evaluated in this study (Table 1). The LiPP benchmark set can be separated into two subsets: a test set consisting of PDB structures released after the cutoff date and a precutoff set consisting of structures released before the cutoff. Among the 331 lipid–protein pairs, 36 complexes were released after the latest training or calibration cutoff date used by any of the tools evaluated in this study (Table 1). These complexes were designated as the LiPP test set to evaluate method performance on unseen data (defined as data not explicitly used for training/calibration). The other 295 lipid–protein pairs comprise the precutoff set.

**Table 1:** Information on the PDB Structures Used for Training or Calibrating the Five Molecular Docking and Co-Folding Tools Evaluated in this Study^a^ ^101^

Method	Training/Calibration Set
AlphaFold 3	Trained on PDB structures with a training cutoff date **2021–09–30** ^68^
Chai-1	Trained on PDB structures with a training cutoff date of **2021–01–21** ^69^
RoseTTAFold AA	Trained on PDB structures with a training cutoff date of **2020–04–30** ^70^
DiffDock-L	Trained on complexes in ^†^PDBbind before **2019** ^64^
AutoDock Vina	Scoring function was weighted using ^†^PDBbind before **2019** ^51^

a ^†^PDBbind only includes drug-like small molecules with drug-likeness (*QED > 0.2), which excludes many lipid molecules due to their long aliphatic hydrocarbon chains that often violate drug-like ranges.^100^ *Quantitative Estimate of Drug-likeness (QED) is a measure of drug-likeness based on the molecular properties of the molecule: molecular weight (MW), octanol-water partition coefficient (ALOGP), number of hydrogen bond donors (HBD), number of hydrogen bond acceptors (HBA), molecular polar surface area (PSA), number of rotatable bonds (ROTB), the number of aromatic rings (AROM) and number of structural alerts (ALERTS).^101^

To assess the distributional differences between the LiPP test set (postcutoff) and the precutoff set (potentially seen during training), we analyzed the similarity of lipid–protein pairs across the two sets from the perspective of both the protein and lipid molecules as described below.

### Protein Similarity

Protein structure similarity analyses were performed using the ProteinCartography tool^93^ (commit 41ff35f, available at https://github.com/Arcadia-Science/ProteinCartography). PDB files of all proteins from the LiPP benchmark set were supplied and processed using the clustering workflow (mode: “cluster”; plotting_modes: “pca_umap”) executed by the command snakemake --snakefile Snakefile --configfile <file-path>/config.yml --use-conda --cores 12. The pipeline computes a pairwise structural similarity matrix based on global protein structure comparisons and performs dimensionality reduction (PCA followed by UMAP) and clustering using the Leiden algorithm.

### Lipid Similarity

Lipid molecules were represented using RDKit^89^ atom pair fingerprints generated with GetHashedAtomPairFingerprintAsBitVect (nBits = 2048). Lipid similarity was quantified using the Tanimoto coefficient calculated from atom-pair fingerprints.^94^ Pairwise chemical similarity between lipids was computed as the Tanimoto coefficient between fingerprint bit vectors using RDKit’s TanimotoSimilarity function. The Tanimoto coefficient calculates the degree of similarity between the query and the target structures, resulting in a score from 0 (no similarity) to 1 (identical).

### Molecular Docking and Structure Prediction Protocols

Co-folding tools (AlphaFold 3, Chai-1, RoseTTAFold AA) were provided with a protein amino acid sequence and a lipid identifier (i.e., SMILES string or CCD ID) as inputs. The software were tasked with predicting lipid–protein complex structures. In contrast, molecular docking tools, such as AutoDock Vina and DiffDock-L, were provided experimental atomic coordinates of protein and lipid atoms, and tasked with predicting the relative position of the lipid. AutoDock Vina was tested using pocket positions inferred from the experimental complexes, whereas DiffDock-L was tested without predefined pocket information. To reflect practical, out-of-the-box usage scenarios, all software were evaluated under default parameters and settings unless otherwise specified, and no additional refinement was performed following their predictions. The workflows and default parameters are described in detail below. All experiments were performed on Georgia Tech’s PACE Phoenix high performance computing cluster. The top poses or structures were retained for subsequent analyses as described below.

### AlphaFold 3

AlphaFold 3 models were generated using a locally installed version of the software (version 3.0.0, commit e2afc41) obtained from the publicly available repository (https://github.com/google-deepmind/alphafold3). AlphaFold 3 model parameters were provided by Google DeepMind for noncommercial use and downloaded on 2024–11–13. For each complex, a JSON input file was prepared containing the protein sequence and the lipid CCD identifier. Structure prediction inference was conducted using the run_alphafold.py script from AlphaFold 3 under default parameter settings. The diffusion-based generative module was executed with 200 denoising steps per sampling trajectory. Iterative refinement of model representations was performed with a recycling depth of 10 (num_recycles = 10). Predictions were computed using a single random seed (modelSeeds = 10). For each input, five independent diffusion trajectories were generated (num_diffusion_samples = 5). The resulting five structural models generated per sample were subsequently ranked according to the model ipTM score and the top scoring model (high ipTM score) was selected for further analysis. All predictions were executed on compute nodes equipped with NVIDIA A100 GPUs, 8 CPU cores, and 48 GB RAM.

### Chai-1

Chai-1 version 0.2.0 was installed as a Python package following the instructions provided in the official GitHub repository (https://github.com/chaidiscovery/chai-lab) using the command pip install chai_lab==0.2.0. To generate a model for each complex, a FASTA file containing the protein sequence and the SMILES string of the lipid was prepared as input for Chai-1. Structure prediction inference was conducted using the run_inference function in predict_structure.py with default inference settings: ESM embedding was enabled (use_esm_embeddings = True). The diffusion-based generative module was executed with 200 denoising steps per sampling trajectory (num_diffn_timesteps = 200). Iterative refinement of model representations was performed with a recycling depth of 3 (num_trunk_recycles = 3). Predictions were computed using a single random seed (seed = 42). For each input, five diffusion trajectories were generated (num_samples = 5). The resulting five structural models generated per sample were subsequently ranked according to the model ipTM score and the top scoring model (highest ipTM score) was selected for evaluation. All experiments were performed on computing nodes equipped with an NVIDIA A100 GPU, 8 CPU cores, and 32 GB of RAM.

### RoseTTAFold AA

RoseTTAFold All-Atom (RoseTTAFold AA) commit 6c85140 was installed following the instructions provided in the official GitHub repository (https://github.com/baker-laboratory/RoseTTAFold-All-Atom). For each complex, a FASTA file containing the protein sequence was prepared, along with a YAML configuration file specifying the FASTA file path and the SMILES string of the lipid. Inference was conducted using the default base.yaml configuration. By default, four recycling iterations were performed (MAXCYCLE = 4). Multiple sequence alignments (MSAs) were limited to 1024 sequences in total (MAXSEQ = 1024), with 128 sequences used in the model’s core MSA stack (MAXLAT = 128). Up to four structural templates (n_templ = 4) were incorporated during inference. One structure was generated per sample using the default inference settings without additional sampling or ensemble averaging. The program was executed with the command python -m rf2aa.run_inference on computing nodes equipped with an NVIDIA A100 GPU, four CPU cores, and 16 GB of RAM.

### AutoDock Vina

AutoDock Vina version 1.2.3 was installed from the official Web site (https://vina.scripps.edu/downloads/). Prior to running AutoDock Vina, atomic coordinates from PDB files from the LiPP benchmark set were converted into AutoDock Vina compatible PDBQT files for the protein and lipid structures separately. Protein structures were processed by removing alternate occupancies and heteroatoms not involved in ligand binding. MGLTools^95^ was used to add polar hydrogens and assign Gasteiger charges to each atom. Lipid structures were standardized by normalizing atom naming conventions, protonation states, and bond orders. In cases where lipids contained alternative atomic positions, only the first coordinate set was retained. The resulting lipid files were converted to PDBQT format using Open Babel version 3.1.1,^96^ which assigned atomic hybridizations, partial charges, and defined rotatable bonds for AutoDock Vina. Binding pockets were inferred from the experimental lipid–protein structures by expanding the lipid’s molecular dimensions 2-fold along all axes to approximate the maximal interaction envelope surrounding the bound lipid. All amino acid residues with any atom located within this expanded volume were identified as close-contact residues defining the binding pocket. Biopython^90^ was used to calculate the geometric centroid of the binding site as the grid box center, and the PyMOL API was used to generate the grid box parameters. After the preprocessing steps, site-specific docking was performed with default parameters (exhaustiveness = 8, number of modes = 9, energy range = 3 kcal/mol). The receptor was treated as rigid, and ligand flexibility was defined by rotatable bonds in the PDBQT files. No fixed random seed was specified. All experiments were executed on computing nodes equipped with an NVIDIA V100 GPU, four CPU cores, and 16 GB of RAM. For each lipid–protein complex, nine poses were generated, and the structure with the most negative (favorable) affinity score (kcal/mol) was selected for further analysis.

### DiffDock-L

DiffDock-L version 1.1.3 was installed from the official GitHub repository (https://github.com/gcorso/DiffDock) following the authors’ instructions. Experimental atomic coordinates (PDB files) of the protein and SMILES strings of the lipids were specified in CSV input files. Default settings from default_inference_args.yaml were used, and the program was executed with the command python -m inference --config default_inference_args.yaml. By default, inference was conducted using 20 diffusion steps (inference_steps = 20) with an exponential noise schedule (expbeta) used during reverse diffusion (sigma_schedule: expbeta). For each complex, 10 poses were generated (samples_per_complex: 10). Stochastic sampling was used (no_random: false), with the final step performed without added noise (no_final_step_noise: true). For each lipid–protein complex, ten poses were generated, and the structure with the best confidence score (highest value) was selected for further analysis. All experiments were performed on computing nodes equipped with an NVIDIA V100 GPU, one CPU core, and 16 GB of RAM.

### Physically Plausibility of Modeled Lipid–Protein Complexes

The PoseBusters test suite was installed following the instructions from the GitHub repository (https://github.com/maabuu/posebusters).^72^ Lipid files were assigned the correct bond orders from PDB Ligand Expo prior to analysis to ensure consistency across methods and maintain uniform conditions for the subsequent physical validity tests. Modeled lipid–protein complexes from all methods were then evaluated using the PoseBusters test suite with the redock configuration. The validity categories are defined as chemical validity, intramolecular validity, and intermolecular validity where (1) chemical validity includes file loading, sanitization, molecular formula, and bond correctness, (2) intramolecular validity includes double bond stereochemistry, tetrahedral chirality, bond lengths, bond angles, internal steric clashes, planarity of double bonds, and energy ratio tests, and (3) intermolecular validity includes minimum protein–ligand distance and volume overlap with the protein. A generated model must pass all tests within a given category to be considered valid for that category. A model that passed all categories is referred to as PB-valid (for PoseBusters test suite valid).

### Comparison of Modeled and Native Lipid–Protein Complex Structures

For models obtained using cofolding methods, the predicted complexes were superimposed onto the experimental structures based on the protein coordinates using the PyMOL API.^91^ This was not performed for molecular docking methods since the PDB file of the protein was used as an input for docking rather than generated by the software. Modeled lipid poses from all methods were compared with the experimental lipid poses by calculating the symmetry-corrected RMSD using spyRMSD.^97^ Prediction success rates were defined as the percentage of cases with lipid RMSD values below a specified cutoff (ranging from 2 to 3 Å) for each method. In a more stringent definition, successful predictions were additionally required to satisfy physical validity criteria (PB-valid) beyond the RMSD threshold. We further analyzed these success rates by grouping the data according to lipid molecular weights and classes, using annotations from the BioDolphin database.

### Evaluation of Scoring Power

We used the following scoring metrics from each method as estimators of the prediction accuracy: ipTM scores for AlphaFold 3 and Chai-1, inter-PAE scores for RoseTTAFold AA, confidence scores for DiffDock-L, and affinity scores for AutoDock Vina. For each complex, the score of the top-ranked model and its lipid RMSD relative to the ground truth experimental structure was obtained for analysis.

### Protein Structure Errors

For each lipid–protein complex modeled by structure prediction methods, we calculated the TM-score and the RMSD score between the experimental and modeled protein structures using the ost compare-structures command from OpenStructure version 2.11.1.^98^ The pTM and ipTM scores were obtained from the top scoring models generated from AlphaFold 3 and Chai-1.

## Results

### Curation of the LiPP Benchmark Set for Modeling Lipid–Protein Interactions

To our knowledge there is no well-curated data set of high-quality lipid–protein structures, limiting the ability to benchmark molecular docking and cofolding tools against lipid–protein binding poses. Inspired by the PoseBusters Benchmark set (small molecule–protein), ProNASet (protein-nucleic acid), and Protein–protein Docking Benchmark v4.0 (protein–protein),^72,76,99^ we developed the Lipid–Protein Poses (LiPP) benchmark set: a curated set of ground truth lipid–protein complex structures for benchmarking, modeling, and fine-tuning algorithms. The LiPP benchmark set was generated by filtering structures obtained from our group’s BioDolphin database,^12^ a comprehensive lipid–protein structure database sourced from the Protein Data Bank (PDB). The details of the filtering process are outlined in the Materials and Methods section and follow a workflow similar to that used to generate the PoseBusters Benchmark set (Supporting Table 1).^72^

The LiPP benchmark set contains a total of 331 nonredundant nonannular high-quality experimental crystal structures of lipid–protein complexes at better than 2 Å resolution. Lipid types represented in LiPP encompass the eight different classes as defined by LIPID MAPS:^8–10^ fatty acyls, glycerolipids, glycerophospholipids, sphingolipids, sterols, prenols, saccharolipids, and polyketides. Fatty acyls, sterols, polyketides, and prenols are the most abundant lipid classes in the data set (Figure 2A). LiPP contains 235 unique lipids, representing an approximately 9% coverage of the lipid universe defined in BioDolphin. We prioritized structural quality over quantity, which necessarily limits the inclusion of certain lipid or lipid–protein interaction types for which high-resolution experimental structures are lacking due to lipid flexibility (low-resolution electron density maps) or challenges in crystallization. The reduction in lipid species primarily results from the requirements of several data filtering steps, including resolution thresholds, structural sanitization issues, steric clashes, and interactions with protein symmetry mates (Supporting Table 1). Most lipid classes in the LiPP data set exhibit greater than 5% coverage relative to BioDolphin (Figure 2B). The underrepresentation of glycerophospholipid and sphingolipid complexes may result from their larger molecular sizes, which increase the likelihood of removal during quality filtering, or their flexibility, which makes atomic-resolution modeling of these lipids in crystal structures difficult. The molecular weights of the lipids in LiPP span from 88 to 1,229 Da (Figure 2C), closely matching the distribution of unique lipid molecules collected in BioDolphin.^12^ Amino acid sequence lengths of the proteins in LiPP show a normal distribution spanning from 107 to 856 residues (Figure 2D). Proteins in LiPP cover a broad range of functional classes, including enzymes, immunological receptors, membrane proteins, signaling receptors, and lipid transport proteins (Figure 2E).

**Figure 2:**
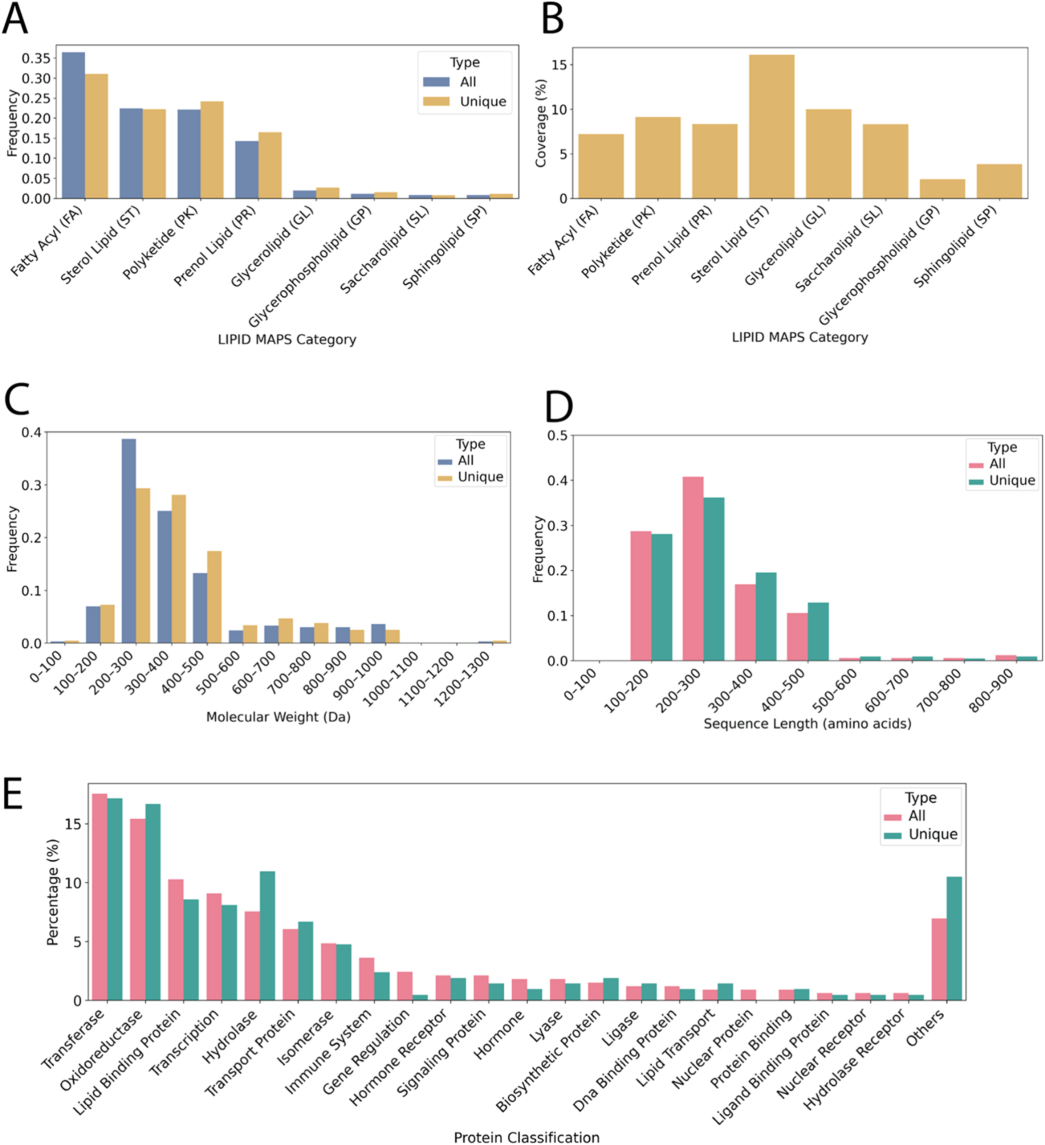
Distributions of lipids and proteins in the LiPP benchmark set. As lipids and proteins may appear more than one time in the data set when they participate in different lipid–protein complex pairs, their statistics can be reported either across all occurrences (“All”) or across unique entries (“Unique”). Lipid uniqueness is defined by the chemical component dictionary (CCD) ID code, whereas protein uniqueness is determined by its cluster (see Methods for details). “Unique”/”All” occurrences are colored in yellow/blue for lipids and green/pink for proteins. (A) Bar plot showing the frequency distribution of lipids in the LiPP benchmark set in each LIPID MAPS category. (B) Bar plot showing the coverage of unique lipids of the LiPP benchmark set within BioDolphin in each LIPID MAPS category. (C) Histogram of lipid molecular weights (Dalton) distribution in the LiPP benchmark set. (D) Histogram of protein sequence lengths (amino acids) distribution in the LiPP benchmark set. (E) Bar plot of the percentages of protein functional classes in the LiPP benchmark set.

Of the lipid–protein complex structures present in the LiPP benchmark set, a total of 36 nonannular lipid–protein pairs were released after 2021–10–01, which represents the date after the most recent training/calibration cutoff date for the five docking and cofolding methods evaluated in this study (Table 1). Motivated to understand how the tools would perform on structures not explicitly seen during training and calibration, we also curated the LiPP “test set” that represents a subset of the structures present in the full benchmark set. Structures present in the LiPP test set were not used for training of AlphaFold 3, Chai-1, RoseTTAFold AA, DiffDock-L, or AutoDock Vina. The statistics of the LiPP test set mimic those in the full benchmark set (Figure 2 and Supporting Figure 2). Together, the features of the LiPP benchmark and test sets suggest that they could serve as a gold standard for modeling lipid–protein interactions. First, they sample a wide range of protein functions and lipid types representative of the diversity of lipid–protein interactome. Second, they include complexes that were not used during training, enabling evaluation on potentially unseen lipid–protein complexes. One obvious caveat is that LiPP does not fully represent the breadth of the lipid–protein interactome, but it is nonetheless an excellent starting point to begin to learn how to model it.

### Physical Plausibility of Docking and Structure Prediction Generated Models of Lipid–Protein Complex

To highlight the utility of LiPP, we performed a benchmark against docking and cofolding tools with the goal of providing the scientific community with guidance on selecting the most appropriate tools for modeling lipid–protein complexes. We evaluated five state-of-the-art molecular docking or cofolding prediction tools: AutoDock Vina,^51^ DiffDock-L,^64^ AlphaFold 3,^68^ Chai-1,^69^ and RoseTTAFold AA.^70^ These software were chosen based on their widespread use in the community, proven performance for protein–protein and small molecule–protein modeling tasks, as well as their open-source availability. Each tool was assessed for the ability to model the 331 nonredundant lipid–protein complexes present in the LiPP benchmark set.

Previous studies suggest that AI-based docking methods often fail to produce physically plausible molecular structures.^72^ Chirality, stereochemical violations, and clashing are especially observed in models produced by cofolding tools.^68–70^ The validity of lipid–protein complex poses generated by both docking and cofolding methods has not yet been systematically evaluated. To address this gap, we first assessed docking or cofolding generated lipid–protein complex structures using the validity checks implemented in the PoseBusters test suite.^72^ There are three categories where a model must pass all tests within a category to be considered valid (herein called PB-valid): chemical validity (file loading, sanitization, molecular formula, and bond correctness), intramolecular validity (stereochemistry, chirality, bond geometry, steric clashes, planarity, and energy ratio), and intermolecular validity (minimum ligand–protein distance and volume overlap).^72^ All software were able to generate chemically valid structures (Figure 3A). Nevertheless, several tools occasionally violated intramolecular and intermolecular validity. Compared to the physics-based method AutoDock Vina, we observed that DiffDock-L exhibited slightly lower passing rates, whereas cofolding methods, such as AlphaFold 3 and Chai-1, maintained validity levels comparable to AutoDock Vina. In contrast, RoseTTAFold AA frequently failed the intermolecular validity tests, particularly the minimum ligand–protein distance test (Figure 3A,B). Incorrect double bond stereochemistry and tetrahedral chirality were common reasons for AI-based methods to fail the intramolecular validity tests (Figure 3B–C). For all methods, maintaining the minimum distance between the lipid and the protein was found to sometimes be difficult (Figure 3B–C), suggesting that additional postprocessing or energy minimization steps may be necessary to resolve steric clashes and improve physical plausibility when modeling lipid–protein complexes. Together, these data suggest that evaluation of model physical plausibility prior to downstream analysis is absolutely necessary to assess the quality of generated lipid–protein complex models.

**Figure 3:**
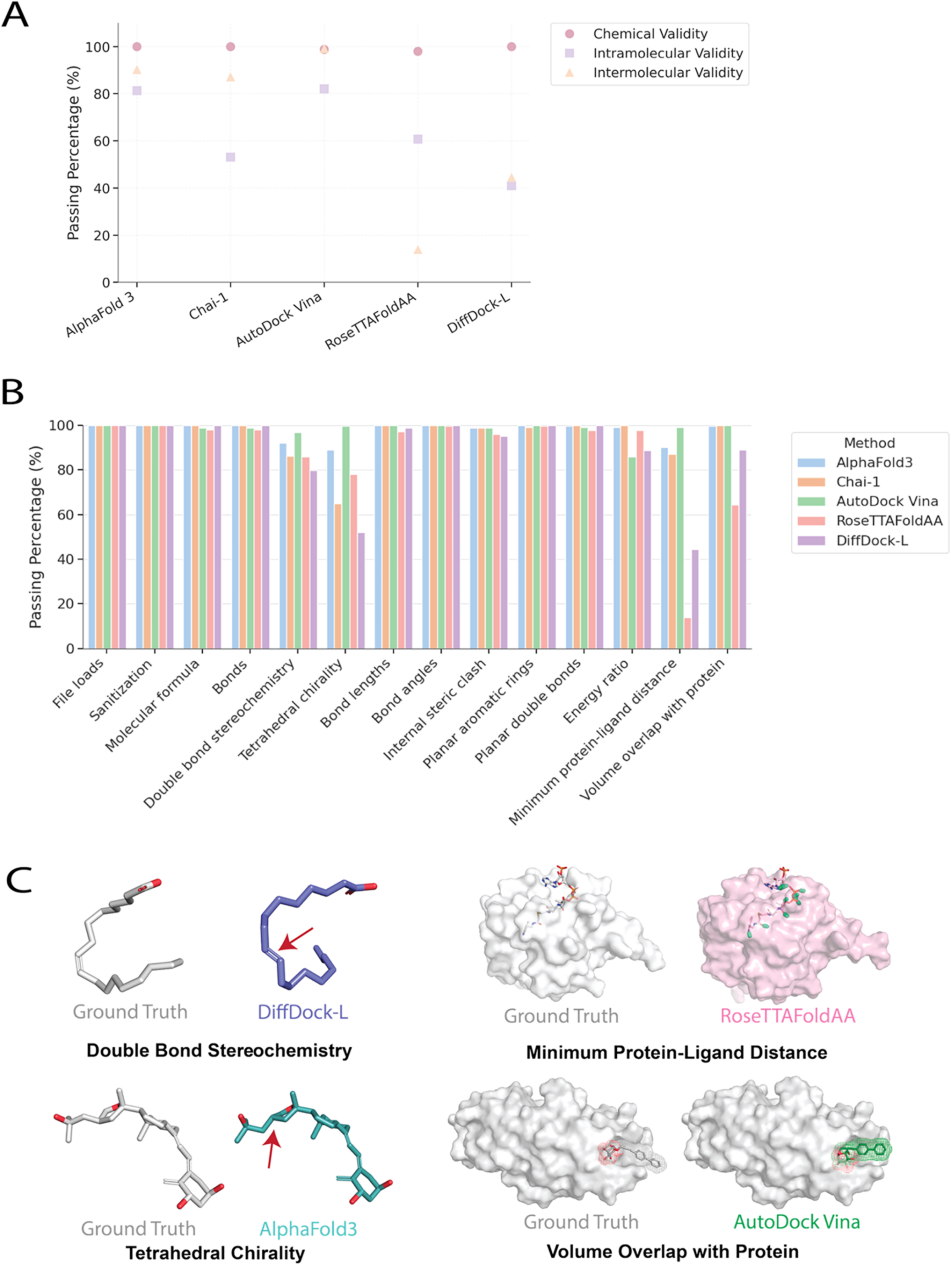
Physical validity tests of generated lipid–protein complex models performed using the PoseBusters test suite. (A) Scatter plot showing the percentage of passing structures for each method on the LiPP benchmark set, categorized into chemical validity, intramolecular validity, and intermolecular validity. A model must pass all tests within a given category to be considered valid for that category. (B) Bar plot comparing all software based on the passing percentages of individual tests in the PoseBusters test suite. (C) Visual examples illustrating common causes of PoseBusters test failures in lipid–protein complexes. Gray structures on the left represent the experimental ground truth lipids or lipid–protein complexes. Examples of validity violations include: (1) Double bond stereochemistry—an oleic acid molecule (BioDolphin ID: BD1hms-A-A-OLA1; PDB ID: 1HMS) predicted by DiffDock-L (purple). (2) Tetrahedral chirality—a lipid with CCD code 0CO (BioDolphin ID: BD3cs6-A-A-0CO1; PDB ID: 3CS6) predicted by AlphaFold 3 (cyan). (3) Minimum protein–ligand distance—a serotonin *N*-acetyltransferase/CoA-s-acetyltryptamine complex predicted by RoseTTAFold AA (BioDolphin ID: BD1cjw-A-A-COT1; PDB ID: 1CJW), where green spheres indicate steric clashes between the lipid and the protein. (4) Volume overlap with protein—a type 1 fimbrin D-mannose-specific adhesin bound to a lipid (CCD ID: FYZ; BioDolphin ID: BD4av5-A-A-FYZ1; PDB ID: 4AV5), with the predicted lipid pose from AutoDock Vina shown in green.

### Modern Computational Methods Are Far from Perfect in Modeling Lipid–Protein Interactions

For each method, we evaluated the top-scoring lipid poses, selected according to each software’s model discrimination criteria, and calculated the all-atom root-mean-square deviation (RMSD) of lipids between the predicted and experimentally determined binding poses. In previous molecular docking and cofold benchmarks of small molecule–protein complexes, RMSD values <2 Å represent the highest quality predictions, values from 2 to 3 Å represent acceptable predictions, and values above 3 Å represent failed predictions.^102,103^ Using these standardized RMSD cutoffs, the success rates among different methods were compared (Figure 4A and Supporting Figure 3A,B and Table 2). Success rates were defined by the percentages of the predicted lipid poses that matched ground truth structures below specific RMSD cutoff values (2 Å, 2.5 Å, 3 Å). AlphaFold 3 (success rate 76.1%) and Chai-1 (60.7%) outperformed the other methods: DiffDock-L (46.8%), AutoDock Vina (47.7%), and RoseTTAFold AA (37.1%) on the LiPP benchmark set. In a stricter definition (PB-valid considered success rates), predictions were considered successful only if they met predefined physical validity requirements in addition to the RMSD threshold of 2 Å. Under this definition, the success rates of all AI methods drop noticeably (Table 3), with AlphaFold 3 (success rate 64.3%), outperforming AutoDock Vina (45.9%), followed by the other methods: Chai-1 (39.5%), DiffDock-L (24.1%) and RosettaFold AA (15.4%). Notably, a similar benchmark of 117 annular lipid–protein interactions not present in the LiPP showed a stark contrast (Supporting Figure 4A,B). Consistent with the fact that annular lipids do not form stable associations with buried protein pockets but instead adopt more variable orientations, none of the five software packages could accurately predict the crystallographically observed positions. Using a lipid RMSD threshold of 2 Å, the success rate was below 15% for each method, and, unlike the results for nonannular lipids, there were no substantial differences in performance across the software.

**Figure 4:**
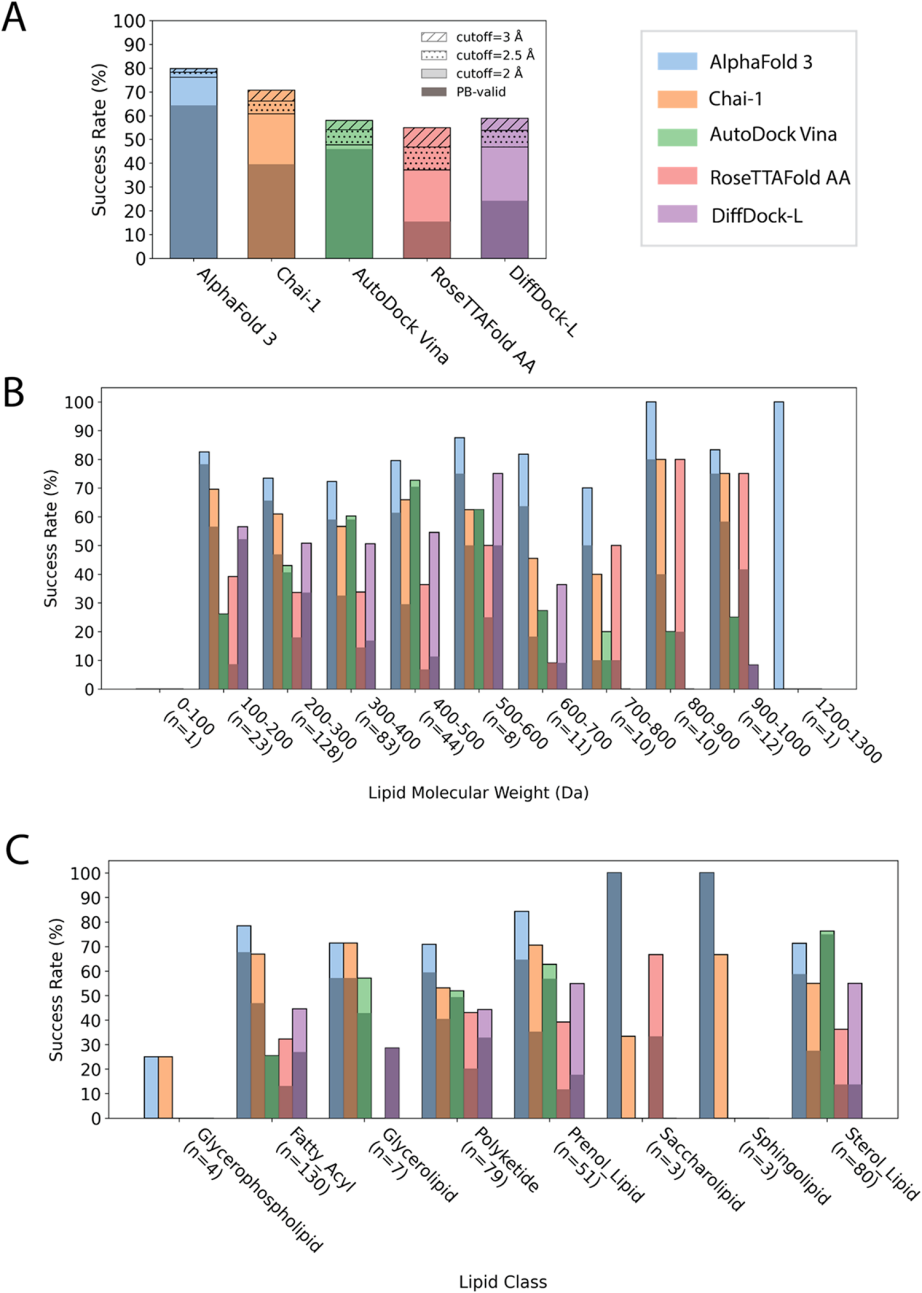
Comparison of success rates of lipid pose predictions across five computational tools on 331 lipid–protein complexes in the LiPP benchmark set. N represents the number of lipid–protein complex data in each subcategories. (A) Bar plot of the success rates obtained from lipid pose all-atom RMSD values of generated models relative to the native experimental structure using RMSD cutoff values of 2 Å, 2.5 Å, or 3 Å. (B) Bar plot of the success rates grouped by different lipid molecular weight using lipid pose all-atom RMSD cutoff values of 2 Å. (C) Bar plot of the success rates grouped by the eight lipid classes using lipid pose all-atom RMSD cutoff values of 2 Å. In panels (A–C), success rates are defined as the percentages of predictions with lipid all-atom RMSD values better than (lower value) the specified cutoff. Success rates defined by passing both RMSD cutoff of 2 Å and physical validity tests are labeled in dark shades (referred to as PB-valid).

**Table 2:** Success Rates (Success Defined Only by Lipid Pose All-Atom RMSD Cutoff Values Less Than 2 Å) of the Five Computational Methods Used in This Study on Lipid–Protein Complexes (via LiPP Benchmark Set) Compared to Protein-Small Molecule Complexes (via PoseBusters Benchmark Set)^a^

Method	LiPP (*N* = 331) % Success Rate	LiPP test set (*N* = 36) % Success Rate	PoseBusters v1 (*N* = 428) % Success Rate	PoseBusters v2 (*N* = 308) % Success Rate
AlphaFold 3	76.1 {71.1–80.6}	47.2 {30.4–64.5}	^†^76.4 {72.1–80.3}^68^	^†^80.5 {75.6–84.8}^68^
Chai-1	60.7 {55.2–66.0}	36.1 {20.8–53.7}	77 ^69^	-
RoseTTAFold AA	37.1 {31.9–42.6}	11.1 {3.1–26.0}	42 ^70^	-
DiffDock-L	46.8 {41.3–52.3}	30.5 {16.3–48.1}	-	50 ^64^
AutoDock Vina	47.7 {42.2–53.2}	30.5 {16.3–48.1}	-	60 ^72^

a *N* denotes the total number of lipid–protein complexes in each dataset. Results of the PoseBusters datasets were extracted from the literature. Values in the brackets denote lower and upper bounds of the 95% confidence intervals calculated with exact binomial distribution. ^†^: Success rates reported by the AlphaFold 3 authors used a cutoff date of 2019-09-30 for training. Success rates of some software has not yet been evaluated on the PoseBusters Benchmark set (denoted with -).

**Table 3:** PB-Valid Considered Success Rates (Success Defined by Passing Both the Criteria of Lipid Pose All-Atom RMSD Cutoff Values Less Than 2 Å and Being PoseBuster Valid) of the Five Computational Methods Used in This Study on Lipid–Protein Complexes (via LiPP) Compared to Protein-Small Molecule Complexes (via PoseBusters)^a^

Method	LiPP (*N* = 331) % Success Rate	LiPP test set (*N* = 36) % Success Rate	PoseBusters v1(*N* = 428) % Success Rate	PoseBusters v2 (*N* = 308) % Success Rate
AlphaFold 3	64.3 {58.9–69.5}	25.0 {12.1–42.2}	-	-
Chai-1	39.5 {34.2–45.0}	16.6 {6.3–32.8}	-	-
RoseTTAFold AA	15.4 {11.6–19.7}	2.7 {0.07–14.5}	-	-
DiffDock-L	24.1 {19.6–29.1}	8.3 {1.7–22.4}	-	-
AutoDock Vina	45.9 {40.4–51.4}	30.5 {16.3–48.1}	-	58 ^72^

a *N* denotes the total number of complexes in each dataset.

We next asked whether the lipid molecular weight influences prediction accuracy. Across lipid molecular weight ranges, the methods exhibited similar trends where AlphaFold 3 and Chai-1 generally perform better than other evaluated tools (Figure 4B). We observed that lipids between 200 and 600 Da showed consistent prediction accuracy for each method. However, larger lipids (>600 Da) were challenging to predict for docking methods, including AutoDock Vina and DiffDock-L, likely due to the exclusion of long aliphatic chains in their training/calibration data sets (Table 1). We also compared the performance of docking and cofolding software across different lipid classes defined by LIPID MAPS.^8–10^ This analysis implies that glycerophospholipids could be more challenging types of lipids to predict for all methods, potentially due to their size and flexibility (Figure 4C). One obvious limitation is the relatively small number of entries for these classes of complexes present in LiPP, making the generalizability of these observations uncertain. In contrast, more rigid, small molecule-like lipid types like sterols, fatty acyls, prenols, and polyketides were modeled with greater accuracy on average (Figure 4C).

A representative example of the C8PhF sphingolipid antigen bound to the CD1d immunoreceptor highlights differences in the performance of the different software against the ground truth CD1d-C8PhF crystal structure (Figure 5).^5^ If the orientation of the C8PhF sphingolipid antigen within the CD1d groove is modeled incorrectly, the resulting interpretation of the biological response may also be flawed, particularly with respect to how the T cell receptor recognizes the polar lipid headgroup. AlphaFold 3, Chai-1, and DiffDock-L were able to place C8PhF within the CD1d groove with the correct orientation while RosettaFold AA and AutoDock Vina incorrectly flipped the lipid relative to the ground truth pose. Additional examples are shown in Supporting Figure 5. Together, these results suggest that while both AI- and physics-based docking and cofolding methods can model certain lipid–protein complexes with high confidence, they exhibit significant limitations in predicting lipid–protein structures more broadly. Accordingly, caution should be exercised when modeling lipid–protein interactions, as prediction accuracy depends strongly on lipid size and class, with current software performing best for small molecule–like lipids. Among the tested methods, the cofolding tools AlphaFold 3 and Chai-1 appear to be the most robust for modeling lipid–protein complexes, showing relatively consistent performance across different lipid classes in both structural accuracy and pose validity.

**Figure 5:**
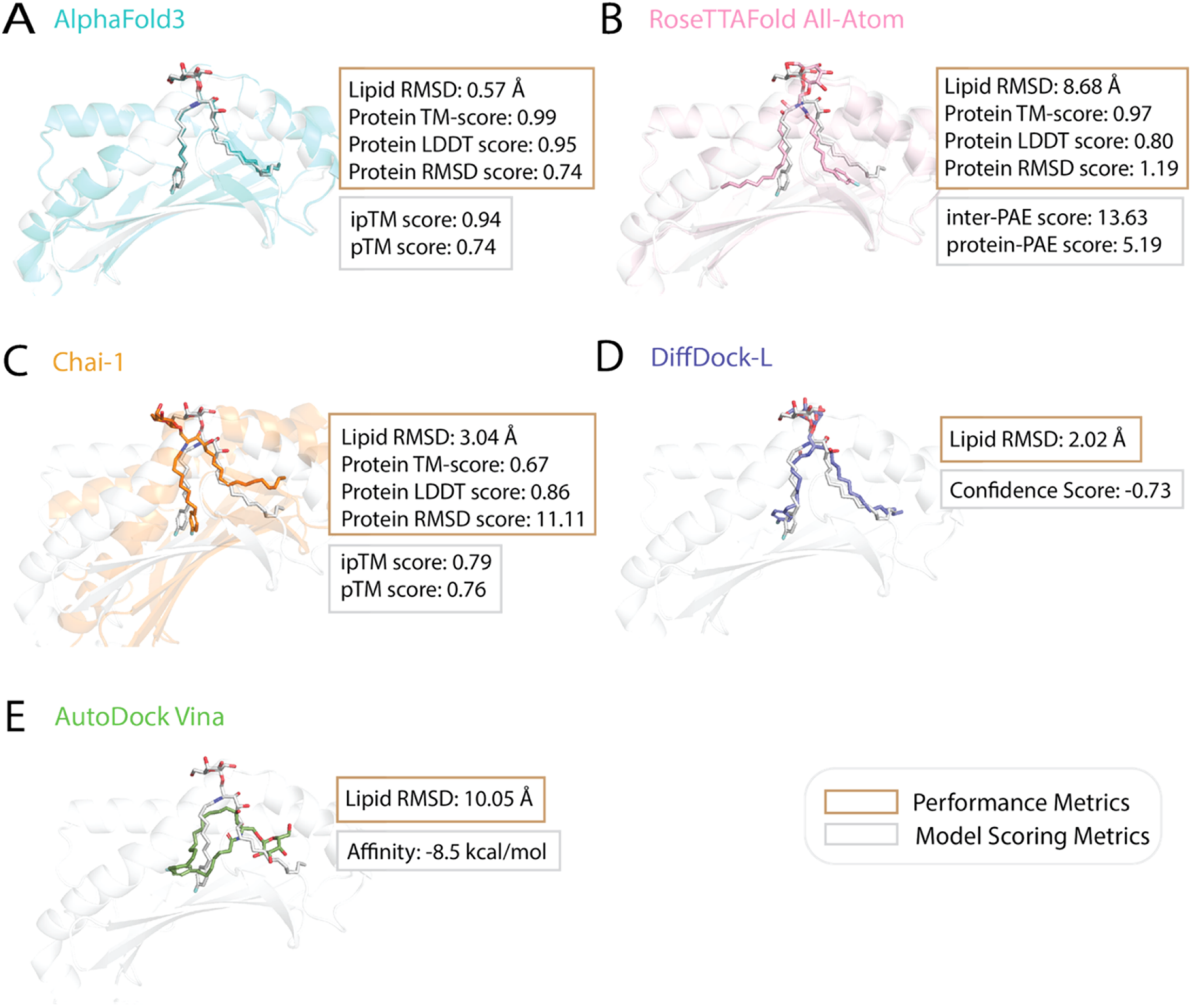
Examples of models obtained for the C8PhF sphingolipid antigen bound to mouse CD1d protein. PDB ID: 3GMO; BioDolphin ID: BD3gmo-A-A-C8F1. The ground truth X-ray structure is shown in gray (lipid in sticks, protein in cartoon). The structure was released before the latest cutoff date and may therefore have been included in the training data of some models; examples of models released after the cutoff with different lipid classes are detailed in the Supporting Information. (A) Lipid pose predicted by AlphaFold 3 (cyan), closely reproduces the ground truth conformation. (B) Lipid pose predicted by RoseTTAFold AA (pink), with the headgroup oriented correctly but the two acyl chains positioned in opposite orientation (∼180° rotation) relative to the ground truth conformation. (C) Lipid poses predicted by Chai-1 (orange) correctly positioned within the binding pocket but showing slight deviations in orientation relative to the ground truth conformation. (D) Lipid poses predicted by DiffDock-L (purple) also correctly positioned within the binding pocket with slight deviations in orientation relative to the ground truth conformation. (E) Lipid pose predicted by AutoDock Vina (green), in which the lipid headgroup is misplaced within the CD1d binding groove relative to the ground truth conformation. In panels (A–E), the Lipid RMSD values represent lipid pose all-atom RMSD values of the models relative to the native pose. The protein scores (TM-score and RMSD score) of structure prediction tools represent the scores of the modeled protein structure compared to the native protein. Confidence metrics for each software are also provided.

### Comparison of the Performance of Docking and Cofolding Tools on Structures before and after Training Cutoff

To evaluate whether AlphaFold 3 and other tools have simply learned to model the lipid–protein complexes they observed during training, we also compared prediction results for a subset of LiPP (the LiPP test set) that is composed of 36 lipid–protein complexes unseen by all methods (Figure 6A and Supporting Figure 2B and Tables 2 and 3). Unsurprisingly, compared to the full LiPP benchmark set (Figure 4A), the performance across methods drops, demonstrating that generalizability remains a key challenge for AI methods. Despite decreased performance, AlphaFold 3 (success rates of 47.2%) remained the most accurate methods, followed by Chai-1 (36.1%), AutoDock Vina (30.5%) DiffDock-L (30.5%), and RoseTTAFold AA (11.1%) (Figure 6A and Table 2). Compared to their capabilities of predicting the interactions between small molecules and proteins, these methods are still far from perfect in predicting lipid–protein interactions. As a relative example: Buttenschoen et al. introduced the PoseBusters Benchmark set of high-quality, drug-like ligand–protein crystal complexes released since 2021.^72^ AlphaFold 3 achieved a success rate of up to 80.5% on the PoseBusters Benchmark set; however, its performance on unseen lipid–protein complexes in the LiPP test set was substantially lower (47.2%) (Table 2). Other software tools show similar trends. Taken together, these data suggest that one reason for the inability of AI- and physics-based software to model lipid binding poses could be due to incomplete training on lipid–protein interactions. Furthermore, when evaluated under a stricter definition that requires both pose validity and an RMSD below 2 Å, the success rates of all AI-based methods decrease substantially (Table 3). Under this criterion, AlphaFold 3 (25%) performs comparably to AutoDock Vina (30.5%), followed by Chai-1 (16.6%), DiffDock-L (8.3%), and RoseTTAFold AA (2.7%). These results suggest that the physics-based approach provides more consistent preservation of physically valid interactions and appears less sensitive to distributional shifts in newly released, out-of-distribution data.

**Figure 6:**
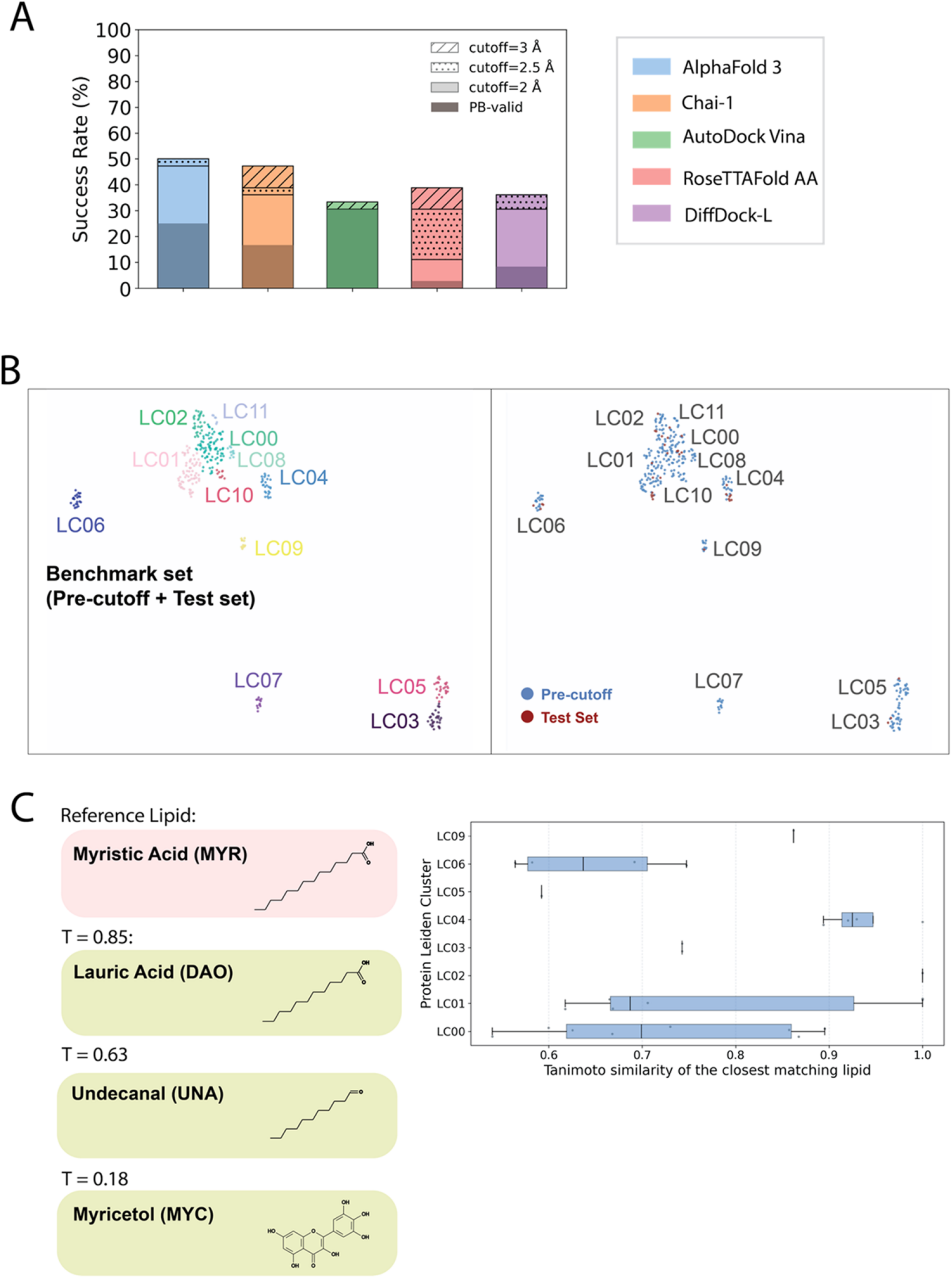
Comparison of success rates of lipid pose predictions across five computational tools on the LiPP test set. (A) Success rates of lipid pose predictions on the LiPP test set (a trimmed version of LiPP with *N* = 36 nonredundant lipid–protein complexes), which only includes complexes released after the training cutoff date for the five computational methods. The bar plot shows success rates defined by lipid pose all-atom RMSD cutoff values of 2 Å, 2.5 Å, and 3 Å, respectively. Success rates defined by satisfying both the 2 Å RMSD cutoff and physical validity criteria are shown in darker shades (PB-valid). (B) Visualization of protein clusters for all complexes in the LiPP benchmark set. LC denotes the protein Leiden clusters. (*Left*) Complexes are colored by according to their assigned Leiden clusters. (*Right*) Complexes are colored their data set: precutoff data are shown in blue, and test set data are shown in red. (C) (*Left*) Example comparison of chemical structures between a reference lipid (myristic acid) and similar/unsimilar lipids (lauric acid, undecanal, and myricetol). *T* denotes the Tanimoto coefficient (similarity score) calculated between the reference lipid and each comparison lipid. (*Right*) Box plot showing the distribution of Tanimoto coefficient for the closest matching lipid in the precutoff set for each test set complex, stratified by their protein clusters.

The full LiPP benchmark data set can be separated into two subsets: the test set described above that consists of PDB structures released after the cutoff date and a precutoff set consisting of structures released before the cutoff. To characterize the distributional differences between the LiPP test set and the precutoff data set, we quantified the similarity of lipid–protein pairs across the two data sets. We first compared the protein folds and protein families present in the LiPP test and precutoff data sets by clustering structures using the ProteinCartography tool.^93^ In cluster mode, ProteinCartography takes as input a set of PDB structures (i.e., all lipid–protein complexes in the LiPP benchmark set), performs an all-versus-all structural comparison using Foldseek to calculate template modeling (TM)-scores, clusters the structures using the Leiden algorithm, and visualizes the resulting clusters with a uniform manifold approximation and projection (UMAP) multivariate analysis plot.^104,105^ The UMAP plots allow us to identify trends in protein family specific structural landscapes across the LiPP benchmark set, test set, and precutoff set. The similarity of protein folds in the lipid–protein complexes among the test set and the precutoff set in LiPP is largely comparable. Specifically, most protein folds present in the LiPP test set fall within the same Leiden clusters as proteins in the precutoff set, with the exceptions of a number of entries in LC07 (FKBP), LC08 (Lipocalins), LC10 (Transcription activator proteins), and LC11 (Transferases) (Figure 6B and Supporting Figure 6). Importantly, however, protein fold-level similarity does not necessarily imply that the corresponding lipid–protein combinations were previously observed during training.

We also evaluated the similarity of lipid structures between the precutoff and test sets. We computed the Tanimoto coefficients (a measure of structural similarity where 1 is a perfect match) between lipids from complexes across the two data sets within the same protein clusters.^94^ For each complex in the test set, stratified by its protein cluster, we report the Tanimoto coefficient to the closest matching lipid in the precutoff set (Figure 6C). The resulting similarity scores varied substantially across Leiden clusters.

For example, complexes in clusters LC02 (mixtures of enzymes), LC04 (FABPs), and LC09 (antigen presenting protein CD1) within the LiPP test set exhibit high lipid similarity with the precutoff lipids, whereas clusters LC00 (mixture of folds), LC01 (mixtures of folds), LC05 (nuclear receptor), and LC06 (CYP P450s) show moderate to low lipid similarity (Figure 6C). We further observe that having highly similar lipid–protein pairs in the precutoff complexes does not necessarily translate into accurate model predictions in the test set (Supporting Figure 7). In several cases, test data containing an identical lipid–protein cluster as those in the precutoff set were still predicted incorrectly, exhibiting large lipid RMSD values (such as LC00 and LC09). In contrast, for the test data in LC03, even though the closest matching lipids were not highly similar, all methods achieved reasonably accurate predictions. These observations raise the possibility that predictive performance may depend not solely on lipid similarity but also on structural features, such as the presence of well-defined binding pockets within certain protein folds. It is possible that some lipid–protein systems form more structurally constrained pockets that are easier to model, whereas others involve fewer specific interactions.

### Comparison of the Discriminatory Power of Model Confidence and Scoring Metrics

We next probed whether each software had any discriminatory power to distinguish between models that were near versus far away from the ground truth lipid binding pose. To this end, we systematically analyzed the correlation between each method’s internal model scoring metric with the lipid RMSD values of its predictions on the LiPP benchmark set (Figure 7). We additionally evaluated these correlations on the subset of predictions that passed the physical validity tests (Supporting Figure 8). Consistent trends are observed across both analyses. Most AlphaFold 3 predictions for lipid–protein complexes are assigned interface predicted template modeling (ipTM) scores of 0.4 to 0.8, representing low to moderately high confidence that the model is correct (Figure 7A and Supporting Figure 8A). While different ranges of ipTM scores exhibit some distinction in lipid RMSD values, predictions with high ipTM scores (>0.8) were also found to yield inaccurate lipid poses. Compared to AlphaFold 3, Chai-1 produces a more even distribution of ipTM scores (Figure 7B and Supporting Figure 8B). High ipTM scores (>0.8) and low ipTM scores (<0.2) are more indicative of prediction accuracy, however, are not perfect as models with low ipTM score exhibit low RMSD values. Interestingly, for RoseTTAFold AA, interchain predicted aligned error (inter-PAE) scores are strongly correlated with lipid RMSD values (Figure 7C and Supporting Figure 8C). Notably, an inter-PAE score above 10 reliably indicates failure in predicting lipid positions, whereas a score below 5 is highly indicative of successful predictions.^66^ For DiffDock-L, a confidence value that is a larger number is better and scores below −1.5 are considered low confidence.^63^ DiffDock-L displays poor correspondence between its confidence scores and lipid pose accuracy, suggesting an inability to distinguish ground truth lipid pose quality (Figure 7D and Supporting Figure 8D). Finally, AutoDock Vina demonstrates a strong relationship between affinity scores and prediction quality (Figure 7E and Supporting Figure 8E). In our benchmark experiments, predictions with affinities below −10 kcal/mol generally correspond to accurate lipid conformations, whereas affinities above 10 kcal/mol are strongly associated with inaccurate lipid poses. Together, these results indicate that AI- and physics-based docking and cofolding software differ substantially in their ability to discriminate correct lipid-binding poses. Among the tested tools, RoseTTAFold AA and AutoDock Vina exhibited the strongest discriminatory power, whereas DiffDock-L performed the poorest.

**Figure 7:**
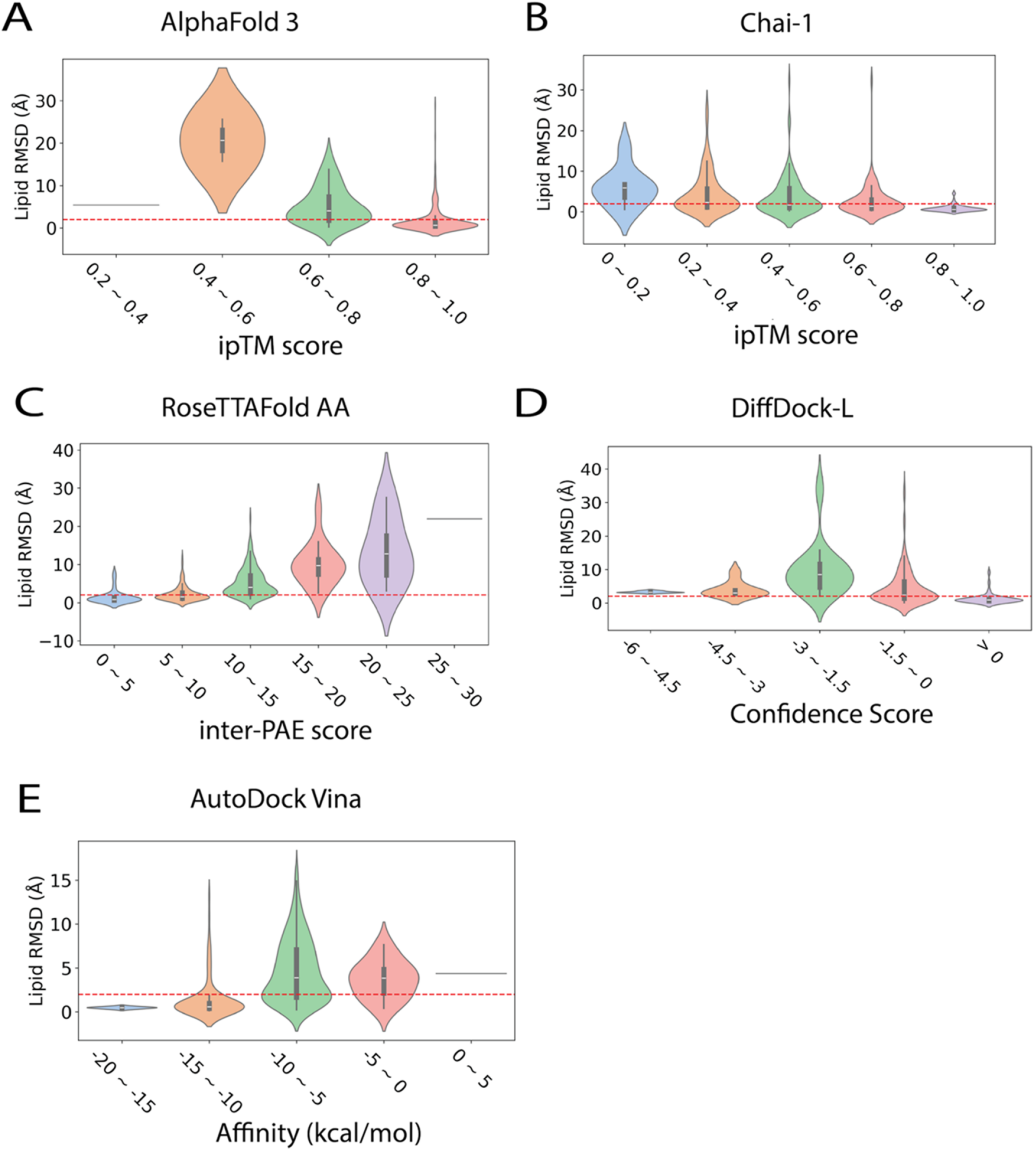
Violin plots highlighting the relationship between each method’s model discrimination score and the corresponding lipid pose all-atom RMSD values. All data in the LiPP data set were included. (A) ipTM scores of AlphaFold 3-based cofolding predictions where higher ipTM represents a more confident prediction. (B) ipTM scores of Chai-1-based cofolding predictions where higher ipTM represents a more confident prediction. (C) Inter-PAE scores of RoseTTAFold AA-based cofolding predictions where lower inter-PAE represents a more confident prediction. (D) Confidence scores of DiffDock-L docking predictions where a higher confidence score represents a more confident prediction. (E) Predicted affinity values (kcal/mol) of AutoDock Vina docking predictions where a more negative value presents a more stable binding pose.

### Protein Prediction Errors in Structure Prediction Methods

Unlike most molecular docking software, cofolding methods predict both the protein and its bound lipid jointly. Thus, we investigated whether lipid binding pose prediction errors primarily arose from inaccuracies in the predicted protein structures. We evaluated the distribution of TM-scores between the predicted and experimental protein structures across the cofolding methods.^106^ Figure 8 presents the correlation analysis for the full LiPP benchmark set. We further evaluated these correlations on the subset of predictions that passed all physical validity tests (Supporting Figure 9). Similar trends are observed across both analyses. All three cofolding approaches achieve high accuracy on the protein monomers in the LiPP benchmark set with lower quartiles of TM-scores exceeding 0.9 across methods (Figure 8A and Supporting Figure 9A). RoseTTAFold AA and Chai-1 exhibit a slightly higher number of outliers with lower TM-scores (<0.6). As expected, in cases where the protein predictions were less accurate, the corresponding lipid poses typically deviated by more than 2 Å RMSD from the experimental lipid structures (Figure 8B–D and Supporting Figure 9B–D). However, even among predictions with highly accurate protein structures, lipid RMSD values vary widely (Figure 8B–D and Supporting Figure 9B–D). These results indicate that for most cases, failure to model the lipid–protein complex is not due to incorrect protein folding, but instead current cofolding methods simply struggle to accurately capture the relative positioning of lipids within lipid–protein complexes.

**Figure 8:**
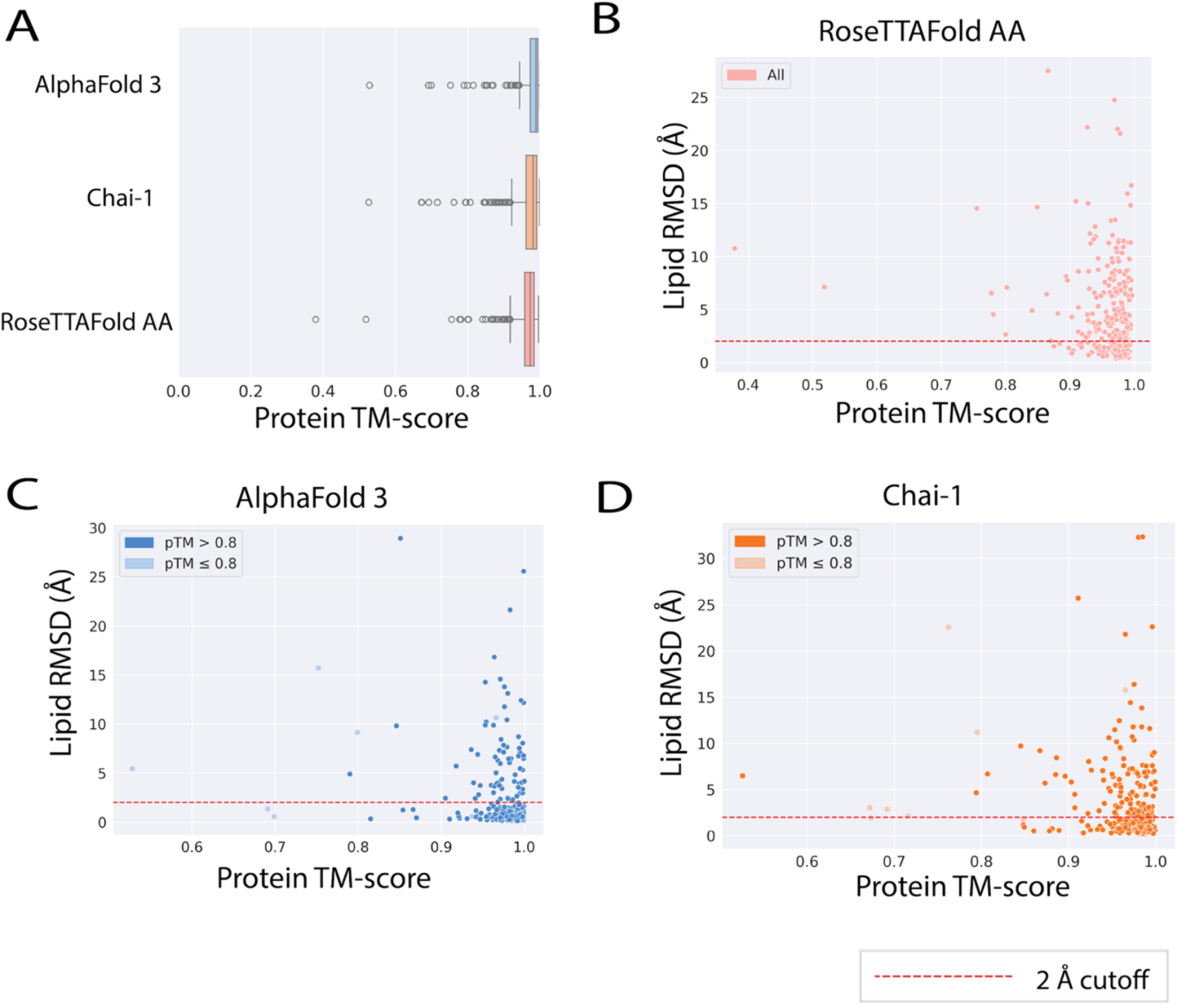
Evaluation of protein structure prediction accuracy in lipid–protein complexes. TM-scores were calculated between the experimental and predicted protein structures. (A) Box plot showing the distribution of protein TM-scores predicted by the structure prediction methods evaluated in this study. (B–D) Scatter plots illustrating the relationship between protein TM-scores and lipid RMSD values. Each point represents a lipid–protein complex in the benchmark set. Scatter plots for AlphaFold 3 and Chai-1 are annotated to distinguish predictions with higher confidence (pTM > 0.8) and lower confidence (pTM ≤ 0.8).

## Discussion

In this study, we developed LiPP, which is to our knowledge the first benchmark set designed to enable systematic evaluation of docking and cofolding prediction methods to model lipid–protein complexes. By assembling a diverse and carefully curated collection of high-quality nonannular lipid–protein structures, LiPP provides a robust and standardized framework for the assessment of lipid–protein structure modeling methods. The data set spans more than 20 protein classifications (over 160 Pfam families) interacting with lipids from eight different LIPID MAPS categories (Figure 2). Using the LiPP benchmark and test sets, we evaluated two docking methods (AutoDock Vina, DiffDock-L) and three cofolding methods (AlphaFold 3, Chai-1, and RoseTTAFold AA) with the aim of providing practical application guidance on how to approach various modeling tasks encompassing the lipid–protein interactome.

Among the evaluated methods, our results demonstrate that AI-based cofolding prediction methods, namely AlphaFold 3 and Chai-1, achieve superior pose accuracy under conventional RMSD-based evaluation criteria (Figure 4A, Table 2, and Figure 6A), likely reflecting the advantage that these foundation models have gained from exposure to broader and more diverse biomolecular structures, including some lipid–protein complexes and other nonstandard ligands. Co-folding approaches may also benefit from inherently capturing induced-fit effects by jointly predicting lipid–protein structures.^55^ However, when physical validity metrics are incorporated and evaluation is restricted to newly released data, performance differences narrow substantially, with AlphaFold 3 and AutoDock Vina showing similar success rates (Figure 6A). These results imply that although AI-based cofolding prediction methods generally achieve higher overall accuracy, physics-based modeling may better consistently preserve physically realistic interactions and demonstrates greater robustness to distributional shifts in unseen data. Future implementation of cofolding methods together with physics-based potentials (i.e., Bolt2-type models), or downstream minimization using molecular dynamics simulations, could combine the strengths of both approaches.^107,108^

Nevertheless, all tested tools show at least some limited ability to accurately capture ground truth lipid–protein interactions. AlphaFold 3 and Chai-1 were previously reported to perform well on protein–protein, protein–nucleic acid, and protein–small molecule assemblies.^68,69^ In LiPP, AlphaFold 3 and Chai-1 perform well across lipids of different molecular weight and lipids of different classes (success rates > ∼70%, Figure 4A–C). There may be substantial challenges in modeling lipid-binding modes for large and complex lipids, such as glycerophospholipids, but the limited number of entries for these types of lipids makes it difficult to be certain (Figure 4C). One possibility is that the high flexibility of lipid molecules, with their many rotatable bonds, creates a vast conformational landscape that existing modeling frameworks do not yet represent well.^109–112^ Another open question is whether lipid–protein interfaces lack the rigid and well-defined binding pockets more frequently seen in other biomolecular complexes, which may inherently exhibit weaker specificity that makes accurate pose prediction more difficult.^83^ Together, these potential factors call for future investigation into the nature of lipid–protein interfaces that will enable improvements in modeling lipid–protein complex structures.

While AlphaFold 3 and Chai-1 provide the most accurate predictions under conventional RMSD-based evaluation for lipid–protein complexes in LiPP, their output models should be interpreted with caution. First, the confidence metrics of cofolding tool outputs may be only partially correlated with the accuracy of lipid poses (Figure 7). Previous studies have shown that ipTM scores are biased by protein sequences, particularly in disordered regions and noninteracting accessory domains,^113^ and that their correlation with prediction accuracy may vary depending on the representativeness of the training data.^114^ Second, these models sometimes generate physically implausible structures especially with clashes between the protein and lipid (Figure 3). As suggested by Errington et al.,^115^ structure prediction methods also often fail to generate plausible poses that preserve meaningful interaction profiles. Combining these shortcomings, ideally, cofolding prediction tools should be paired with physics-based refinement methods, such as energy minimization or molecular dynamics simulations,^15,116^ to ensure more reliable structural modeling.

Using the PoseBusters Benchmarking set, Buttenschoen et al.^72^ concluded that contemporary AI-based docking models do not outperform physics-based tools when assessed on unseen structures released after their training cutoff. It is important to note, however, that the deep learning approaches evaluated in the study were exclusively docking methods. In contrast, recent publications on cofolding architectures report success rates exceeding those of physics-based docking tools when evaluated on the PoseBusters Benchmark set.^73,74^ Together, these observations suggest that cofolding approaches represent a distinct modeling paradigm and may offer improved generalization to previously unseen complexes. A similar pattern is observed in our lipid–protein modeling benchmark: cofolding methods such as AlphaFold 3 and Chai-1 achieve higher RMSD-based pose success rates than both classical physics-based docking and deep learning-based docking approaches on unseen data. However, when success rates are jointly evaluated using both RMSD-based metrics and measures of physical validity on unseen complexes, the performance of all AI-based methods declines substantially, with most falling below that of the physics-based method AutoDock Vina, consistent with the conclusions of the PoseBusters study. These results indicate that improvements in geometric pose accuracy afforded by cofolding methods do not necessarily translate into superior physical plausibility or robust out-of-distribution generalization. Rather, they highlight both the promise of AI-based cofolding structure predictions and the remaining space for improvement, particularly in integrating stronger physicochemical constraints and enhancing robustness to distributional shift.

LiPP represents the first standardized lipid–protein complex benchmarking data sets. As highlighted here, it can enable quantitative assessments of different modeling strategies to capture distinctive features of nonannular lipid–protein interactions. The systematic comparison of different approaches for modeling lipid–protein complexes reveals the advantages and shortcomings of each type of method. These types of benchmarks are essential to illuminate key challenges faced by different tools when modeling lipid–protein interactions, such as physical validity, scoring power, and failure of modeling specific lipid types (Figures 3, 4, and 7). As molecular modeling frameworks become increasingly generalizable, LiPP could be key for enabling comprehensive assessments of biomolecular interactions that extend beyond small molecules, nucleic acids, and proteins. Additionally, it helps shape the next generation of training strategies and methodological improvements to address the complexity of lipid–protein systems.

Our study has several limitations. First, although LiPP spans a broad diversity of protein families and lipid classes (Figure 2), it does not fully capture the entire breadth of the lipid–protein interactome. Because lipid–protein complexes are far less abundant in the PDB than protein–protein or protein–small molecule interactions,^12,117,118^ it is challenging to assemble a large, high-quality lipid–protein benchmark using purely unseen structures. To address this, we report results on both a general benchmark set and a test set of LiPP, each with its own advantages and limitations. The general benchmark set offers broader lipid coverage but may include complexes that some models have seen during training (Figure 2). Nevertheless, given the overall sparsity of lipids in the training data of current prediction models, this set remains useful for assessing whether models generally capture lipid–protein interaction patterns. In contrast, the LiPP test set is limited but contains lipid–protein complexes that are unseen by all models, enabling a more reliable evaluation of generalizability, albeit with reduced lipid coverage (Supporting Figure 2). We also acknowledge that filtering for high-quality lipid–protein structures inevitably generates trade-offs; larger and more complex lipids are more likely to be excluded. Second, our benchmark focused on a subset of widely used and openly accessible modeling tools. Because protein–ligand modeling methodologies are rapidly evolving, we did not evaluate all emerging platforms, such as DynamicBind,^119^ Boltz,^120^ or NeuralPlexer3,^121^ or commercial docking engines like Glide,^52^ GOLD,^53^ or MOE,^122^ which may offer complementary capabilities. However, given that many new methods employ training sources like those tested in this study, we predict their performance on lipid–protein systems would likely not differ substantially. Third, our study focused solely on lipid–protein monomeric complexes (one-to-one stoichiometry), excluding more complex interactions involving multichain assemblies.^123^ This omission could limit the generalizability of our benchmark set to broader biologically relevant systems. Fourth, we focused only on single-state lipid–protein structures. The dynamic conformational landscapes of these complexes, as well as more challenging scenarios were not explicitly evaluated. However, with tools like AF-Cluster, AlphaFlow, and BioEmu, significant effort is being made in predicting conformational landscapes; we expect these tools to be key for enhanced accuracy in modeling lipid–protein interactions.^124–126^ Fifth, here we applied the validity criteria from the PoseBusters test suite originally developed for small molecules to lipid molecules.^72^ It is not clear whether the tool directly translates between different types of molecular systems. However, we expect this is not a major issue because many of the evaluated criteria reflect fundamental physicochemical constraints, such as bond geometry and steric clashes, that are likely general to most molecular systems.

Finally, as this study was designed to reflect practical, out-of-the-box usage scenarios, all software tools were evaluated using default parameters and settings (details in Materials and Methods section). The computational comparison between AI-based methods and AutoDock Vina therefore reflects typical deployment conditions rather than strictly equalized hardware configurations: AI methods were executed on GPU accelerators, whereas Vina was run on CPU resources. Because docking and structure prediction performance can depend on computational allocation and sampling depth, additional resources or parameter tuning could potentially improve accuracy for certain methods. Accordingly, our results should be interpreted as representing practical usage conditions rather than theoretical compute-normalized performance limits. Enhanced sampling and molecular dynamics-based refinement approaches, while powerful, were not included given the defined scope of this benchmark study.

Overall, the study highlights several important avenues for future research. In our view, progress in the field will require: (1) deeper understanding of the binding mechanisms underlying lipid–protein interactions to enable incorporation of lipid-binding domain knowledge into deep learning model architectures and scoring metrics, (2) deployment of AI- or physics-based methods trained or fine-tuned on lipid–protein specific data, and (3) development of models capable of capturing dynamic interaction processes.^55,119,124,125,127^ Together, these efforts will be essential for improving the accuracy and generalizability of lipid–protein structure prediction.

## Conclusions

Recent advances in molecular docking and structure prediction have enabled accurate modeling of ligand-protein structures yet studying lipid–protein systems remains difficult due to a lack of validated methods for these complex molecules. Most docking tools are optimized for small molecules rather than lipids, whose distinct structures challenge their applicability. In this study, we created LiPP, a new gold standard benchmark set of high-quality lipid–protein crystal structures, to systematically evaluate current docking and structure prediction methods for modeling lipid–protein complexes. Our results suggest that current ligand-protein modeling methods, even those based on foundation models trained on diverse biomolecules, remain only partially suited for predicting most lipid–protein complex structures. These findings underscore that accurate modeling of lipid–protein systems remains a significant challenge. The binding modes and mechanisms of lipid–protein complexes may differ substantially from those of small molecule-protein interactions, highlighting the need for future efforts to delineate the distinctive features of lipid–protein binding. Advances in modeling approaches will be essential to capture the unique structural characteristics of lipid–protein interactions.

## Supplementary Material



## Data Availability

The LiPP benchmark/test sets and associated scripts for running the benchmarks are provided on GitHub at https://github.com/mcshanlab/LiPP_Benchmark.

## Notes

Construction, composition, and evaluation of the LiPP benchmark set for lipid–protein complex modeling, including analyses of data set diversity, benchmarking performance across prediction methods, and representative modeling examples, and filtering workflow used to curate the benchmark set (PDF)

## Abbreviations

AI: artificial intelligence
CCD ID: Chemical Component Dictionary ID
RMSD: root-mean-square deviation
pTM: predicted template modeling
SMILES: Simplified Molecular Input Line Entry System
UMAP: Uniform Manifold Approximation and Projection
ipTM: interface predicted template modeling
inter-PAE: interchain Predicted aligned error
TM-score: template modeling score
PDB: RCSB Protein Data Bank
RoseTTAFold All-Atom: RoseTTAFold AA
PB-valid: PoseBusters valid (passed the PoseBusters test suite)
QED: Quantitative Estimate of Drug-likeness
