# Supplementary material for: The LiPP Benchmark Set for Modeling Lipid–Protein Complexes: Comparison of Co-Folding and Docking Methods
Source: J Chem Inf Model. 2026 Jun 10;66(12):7231–51. doi: 10.1021/acs.jcim.6c01457 (PMC13292216; doi:10.1021/acs.jcim.6c01457)
Supplement: Supplementary file 1 [file ci6c01457_si_001.pdf]

Supporting Information for:

The LiPP Benchmark Set for Modeling Lipid–Protein Complexes: Comparison of Co-Folding and Docking Methods

Li-Yen Yang[1], Shreyas Gupta[1,2], Lauren N. Mullininx[1,2], Andrew C. McShan[1*]

[1]School of Chemistry and Biochemistry, Georgia Institute of Technology, Atlanta, GA 30332, USA

[2]School of Biological Sciences, Georgia Institute of Technology, Atlanta, GA 30332, USA

*Correspondence: Andrew C. McShan (andrew.mcshan@chemistry.gatech.edu)

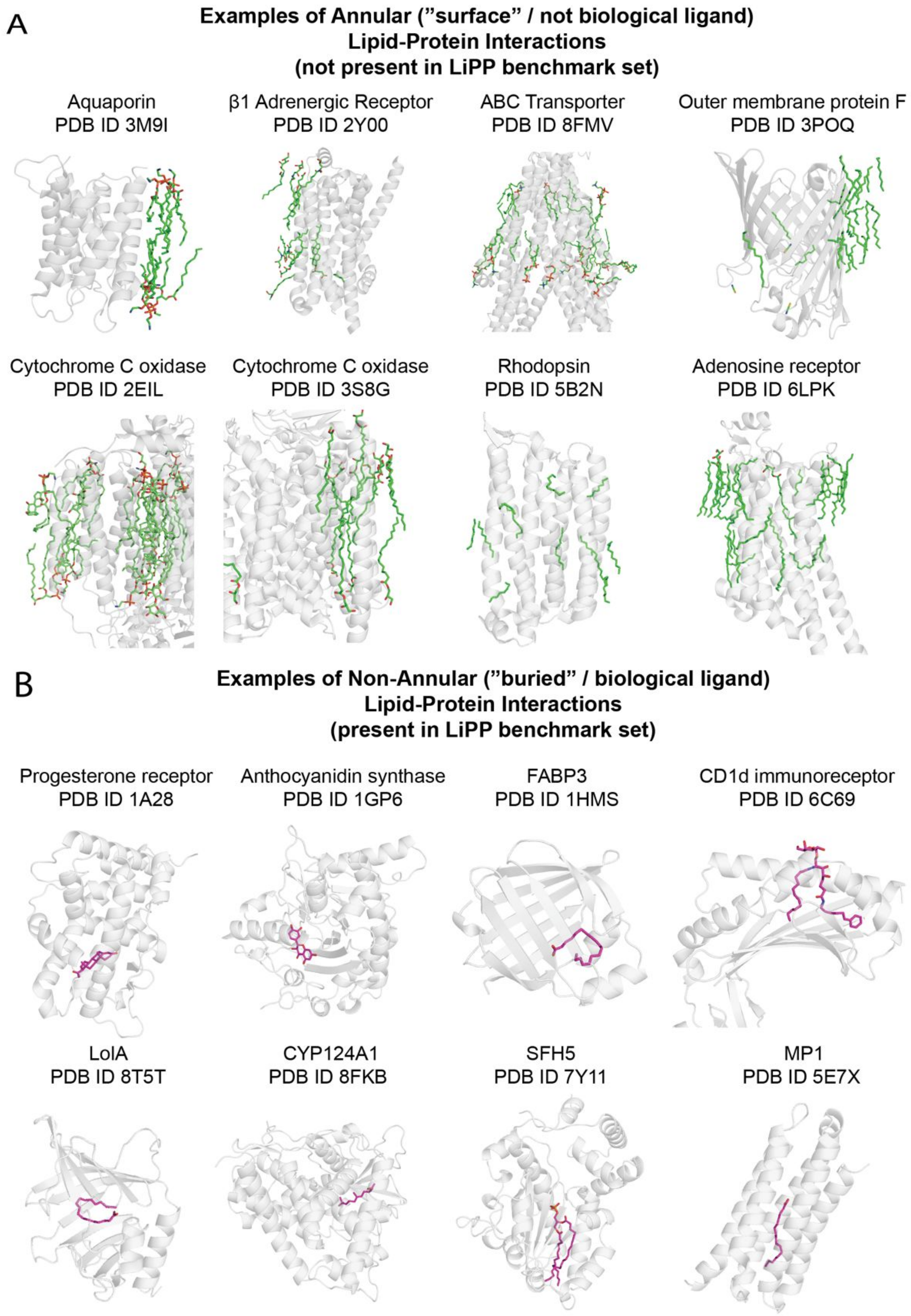

Supplementary Figure 1. Representative examples of **A.** annular (surface, not biological ligands) and **B.** non-annular (buried, biological ligand) lipid-protein interactions. Annular lipids are shown as green sticks. Non-annular lipids are shown as magenta sticks. Proteins are shown as gray cartoon. The PDB ID code for each example is given.

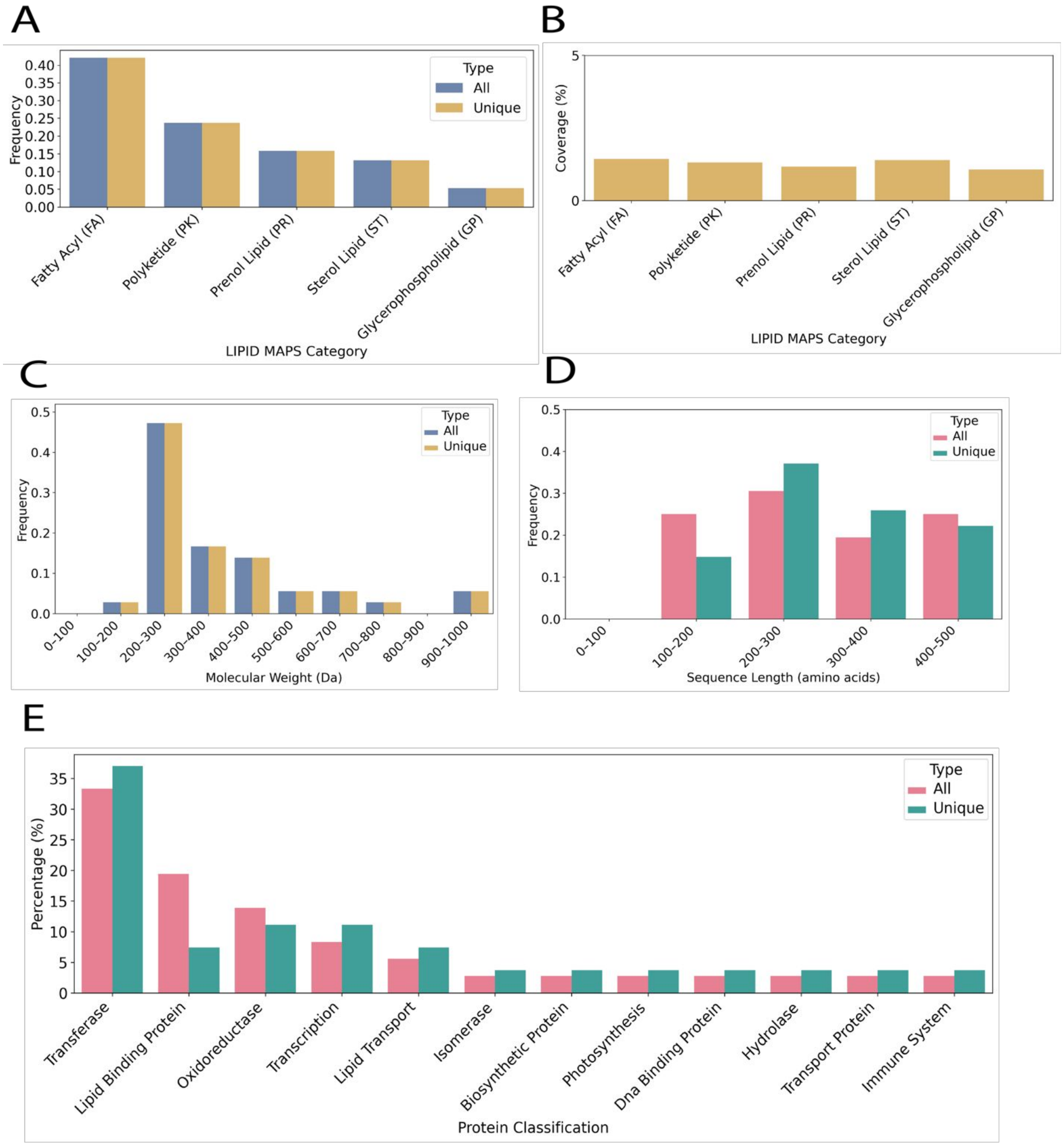

Supplementary Figure 2. Distributions of lipids and proteins in the LiPP test set. As lipids and proteins may appear more than one time in the dataset when they participate in different lipid–protein complex pairs, their statistics can be reported either across all occurrences (“All”) or across unique entries (“Unique”). Lipid uniqueness is defined by the CCD code, whereas protein uniqueness is determined by its cluster (see Methods for details). “Unique”/”All” occurrences are colored in yellow/blue for lipids and green/pink for proteins. **A.** Bar plot showing the frequency distribution of lipids in the LiPP test set in each LIPID MAPS category. **B.** Bar plot showing the coverage of unique lipids in the LiPP test set within BioDolphin for each LIPID MAPS category. **C.** Histogram of lipid molecular

weight (Da) distribution in the LiPP test set. **D.** Histogram of protein sequence length (amino acids) distribution in the LiPP test set. **E.** Bar plot of the percentages of protein functional classes in the LiPP test set.

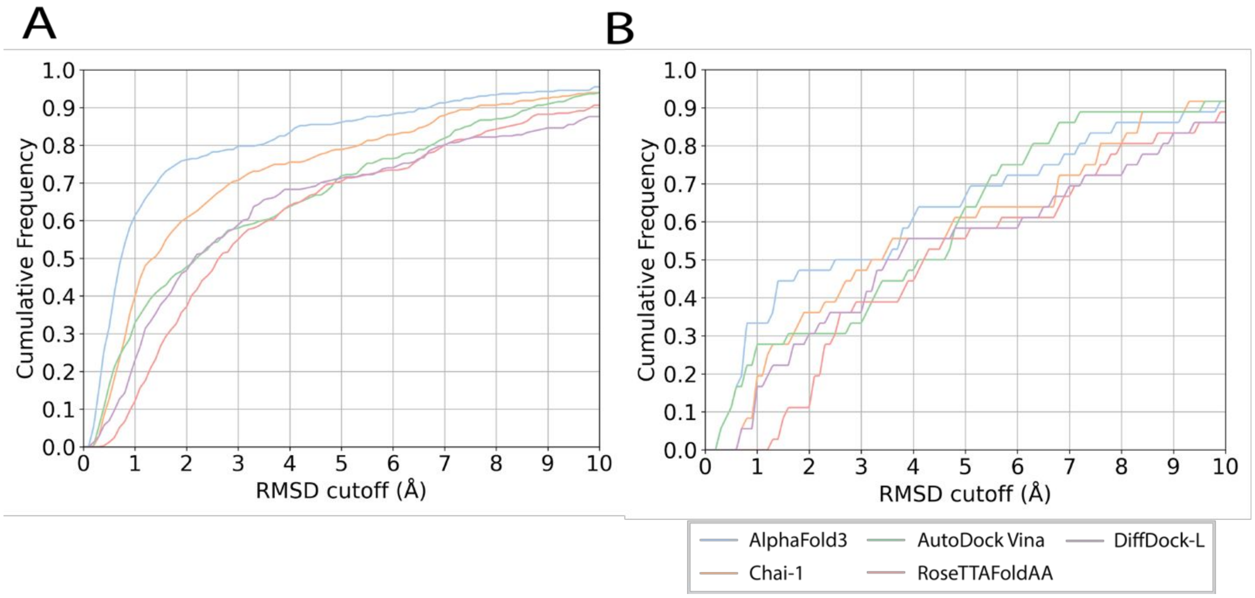

Supplementary Figure 3. Cumulative frequency plots of lipid pose all-atom RMSD values of generated models relative to the native experimental structure using RMSD cutoff values of ranging from 0 to 10 Å. **A.** Cumulative frequency plot on the LiPP benchmarking set. **B.** Cumulative frequency plot on the LiPP test set.

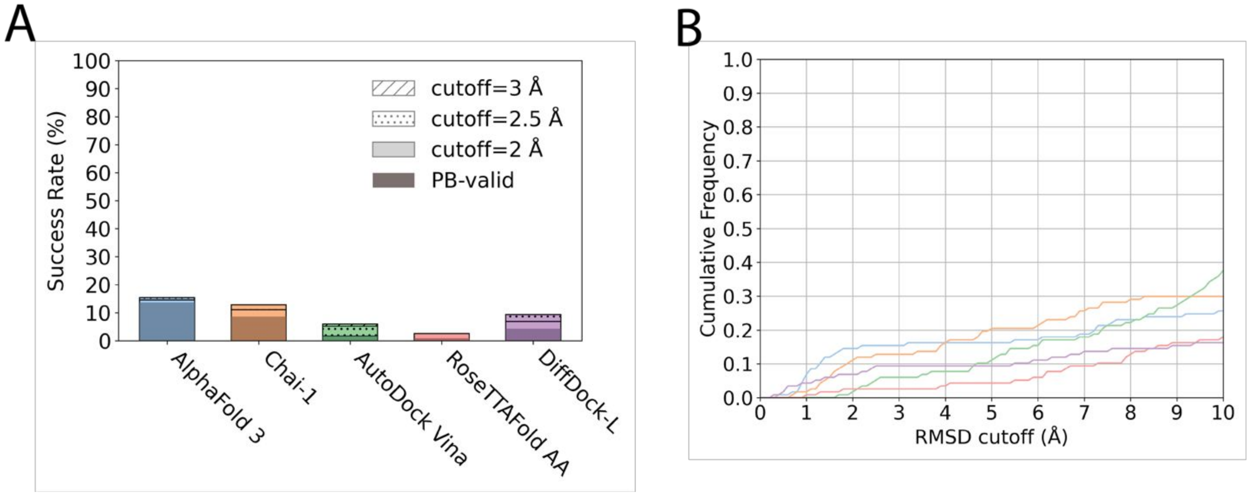

Supplementary Figure 4. Comparison of success rates of lipid pose predictions across five computational tools on 117 annular lipid–protein complexes not present in the LiPP benchmark set. N represents the number of lipid–protein complex data in each subcategories. **A.** Bar plot of the success rates obtained from lipid pose all-atom RMSD values of generated models relative to the native experimental structure using RMSD cutoff values of 2 Å, 2.5 Å, or 3 Å. **B.** Cumulative frequency plot of annular lipid–protein complexes not present in the LiPP benchmark set

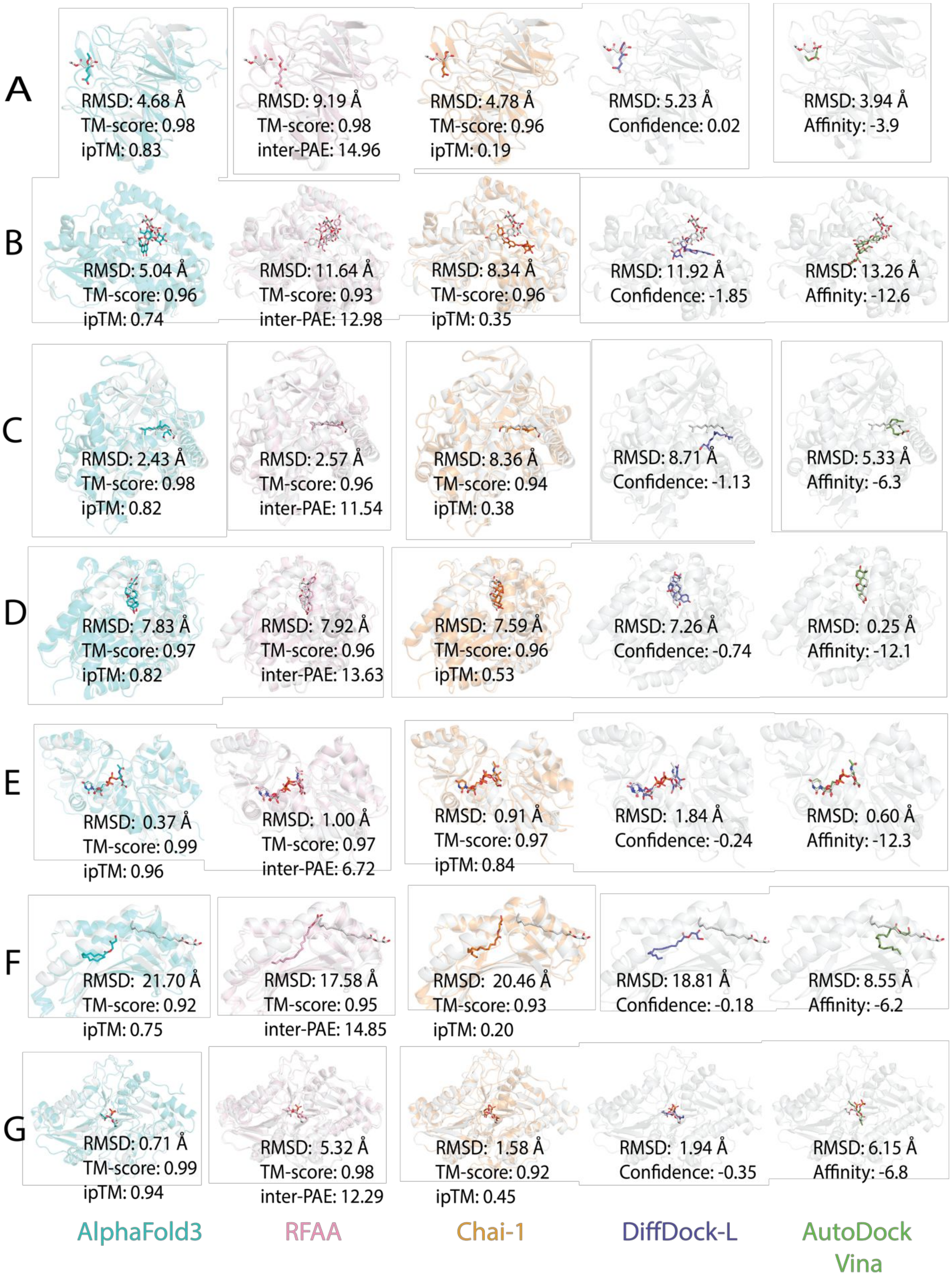
A
RMSD: 4.68 Å
TM-score: 0.98
ipTM: 0.83
RMSD: 9.19 Å
TM-score: 0.98
inter-PAE: 14.96
RMSD: 4.78 Å
TM-score: 0.96
ipTM: 0.19
RMSD: 5.23 Å
Confidence: 0.02
RMSD: 3.94 Å
Affinity: -3.9
B
RMSD: 5.04 Å
TM-score: 0.96
ipTM: 0.74
RMSD: 11.64 Å
TM-score: 0.93
inter-PAE: 12.98
RMSD: 8.34 Å
TM-score: 0.96
ipTM: 0.35
RMSD: 11.92 Å
Confidence: -1.85
RMSD: 13.26 Å
Affinity: -12.6
C
RMSD: 2.43 Å
TM-score: 0.98
ipTM: 0.82
RMSD: 2.57 Å
TM-score: 0.96
inter-PAE: 11.54
RMSD: 8.36 Å
TM-score: 0.94
ipTM: 0.38
RMSD: 8.71 Å
Confidence: -1.13
RMSD: 5.33 Å
Affinity: -6.3
D
RMSD: 7.83 Å
TM-score: 0.97
ipTM: 0.82
RMSD: 7.92 Å
TM-score: 0.96
inter-PAE: 13.63
RMSD: 7.59 Å
TM-score: 0.96
ipTM: 0.53
RMSD: 7.26 Å
Confidence: -0.74
RMSD: 0.25 Å
Affinity: -12.1
E
RMSD: 0.37 Å
TM-score: 0.99
ipTM: 0.96
RMSD: 1.00 Å
TM-score: 0.97
inter-PAE: 6.72
RMSD: 0.91 Å
TM-score: 0.97
ipTM: 0.84
RMSD: 1.84 Å
Confidence: -0.24
RMSD: 0.60 Å
Affinity: -12.3
F
RMSD: 21.70 Å
TM-score: 0.92
ipTM: 0.75
RMSD: 17.58 Å
TM-score: 0.95
inter-PAE: 14.85
RMSD: 20.46 Å
TM-score: 0.93
ipTM: 0.20
RMSD: 18.81 Å
Confidence: -0.18
RMSD: 8.55 Å
Affinity: -6.2
G
RMSD: 0.71 Å
TM-score: 0.99
ipTM: 0.94
RMSD: 5.32 Å
TM-score: 0.98
inter-PAE: 12.29
RMSD: 1.58 Å
TM-score: 0.92
ipTM: 0.45
RMSD: 1.94 Å
Confidence: -0.35
RMSD: 6.15 Å
Affinity: -6.8
AlphaFold3
RFAA
Chai-1
DiffDock-L
AutoDock Vina

Supplementary Figure 5. Examples of modeled lipid–protein complexes with lipids from different LIPID MAPS categories. Modeled complex structures are shown in cyan (AlphaFold 3), pink (RoseTTAFold AA), orange (Chai-1), purple (DiffDock-L), and green (AutoDock Vina). Proteins from structure prediction tools are superimposed with the ground truth X-ray structures shown in gray (lipid in sticks, protein in cartoon). RMSD denotes the lipid pose all-atom RMSD values of the predicted lipid models relative to the native experimental lipid structure. TM-score denotes the TM-score between the predicted protein structures and the native experimental protein structure. Confidence scores (AlphaFold 3 and Chai-l: ipTM score; RoseTTAFold AA: inter-PAE score; DiffDock-L: Confidence score; AutoDock Vina: Affinity in kcal/mol) of the models are also presented. Panels A-E presents examples of structures in the LiPP test set that are unseen by all models. **A.** Fatty Acyl: Monoethyl fumarate bound to Kelch-like ECH-associated protein 1. (PDB ID: 7C60; BioDolphin ID: BD7c60-A-A-NF32) [95]. **B**. Polyketide: Naringin bound to the UDP-glycosyltransferase protein. (PDB ID: 8SFU; BioDolphin ID: BD8sfu-B-B-ZWN1). **C.** Prenol Lipid: Farnesol bound to the Cytochrome P450 protein. PDB ID: 8FKB; BioDolphin ID: BD8fkb-A-A-FOF1 [96]. **D.** Sterol Lipid: Resibufogenin bound to a Glycosyltransferase protein. PDB ID: 8IND; BioDolphin ID: BD8ind-A-A-6JI1 [97]. **E.** Saccharolipid: Uridine-Diphosphate-N-Acetylglucosamine bound to a Glycosyltransferase protein. PDB ID: 8JJT; BioDolphin ID: BD8jjt-C-C-UD11 [98]. **F.** Glycerolipid: Glycerol palmitate antigen bound to a human CD1c protein. PDB ID: 7MX4; BioDolphin ID: BD7mx4-A-A-ZP71 [99]. **G.** Glycerophospholipid: Phosphatidic acid bound to a Phospholipase D protein. PDB ID: 7JRW; BioDolphin ID: BD7jrw-A-A-VHY1 [100].

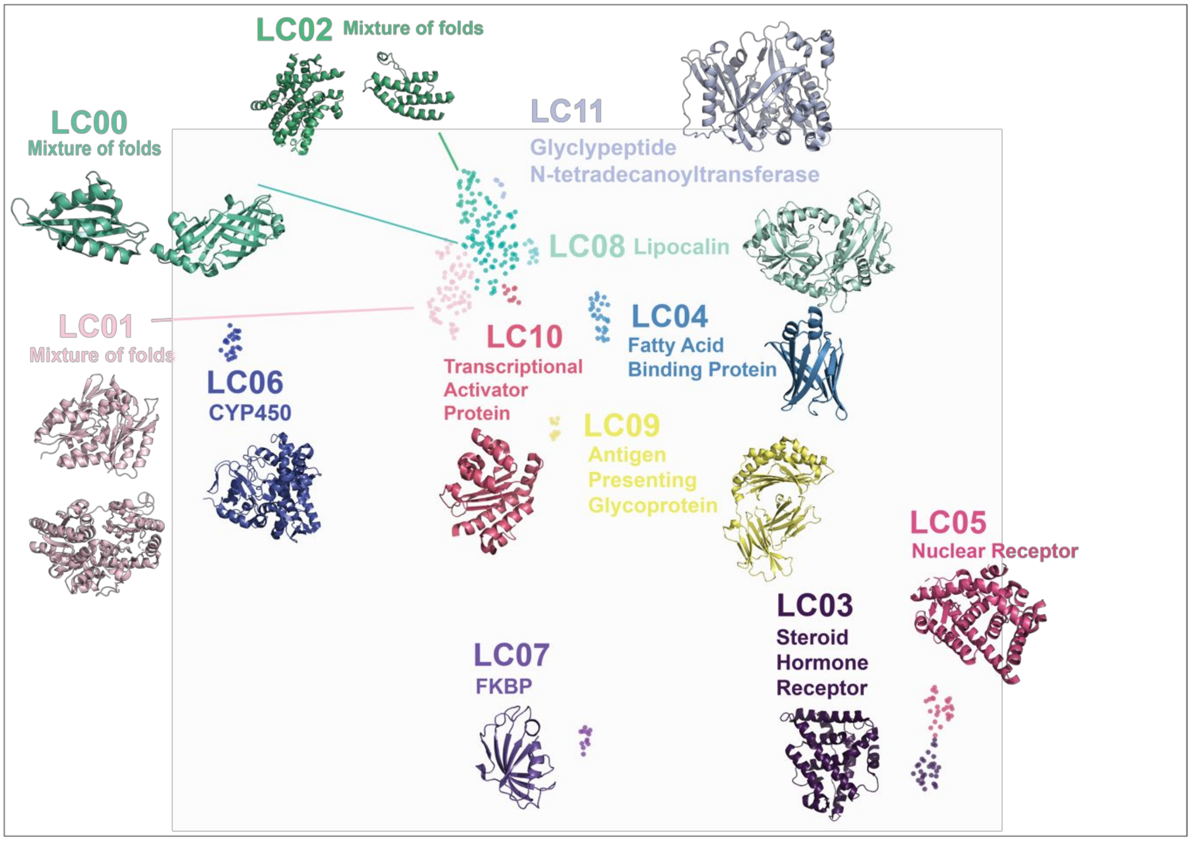

Supplementary Figure 6. UMAP plot of protein Leiden clusters for all complexes in the LiPP benchmark set. LC00–LC11 represent the 12 protein Leiden clusters, annotated with their representative structural folds or protein family names. Generated using ProteinCatrography in cluster mode.

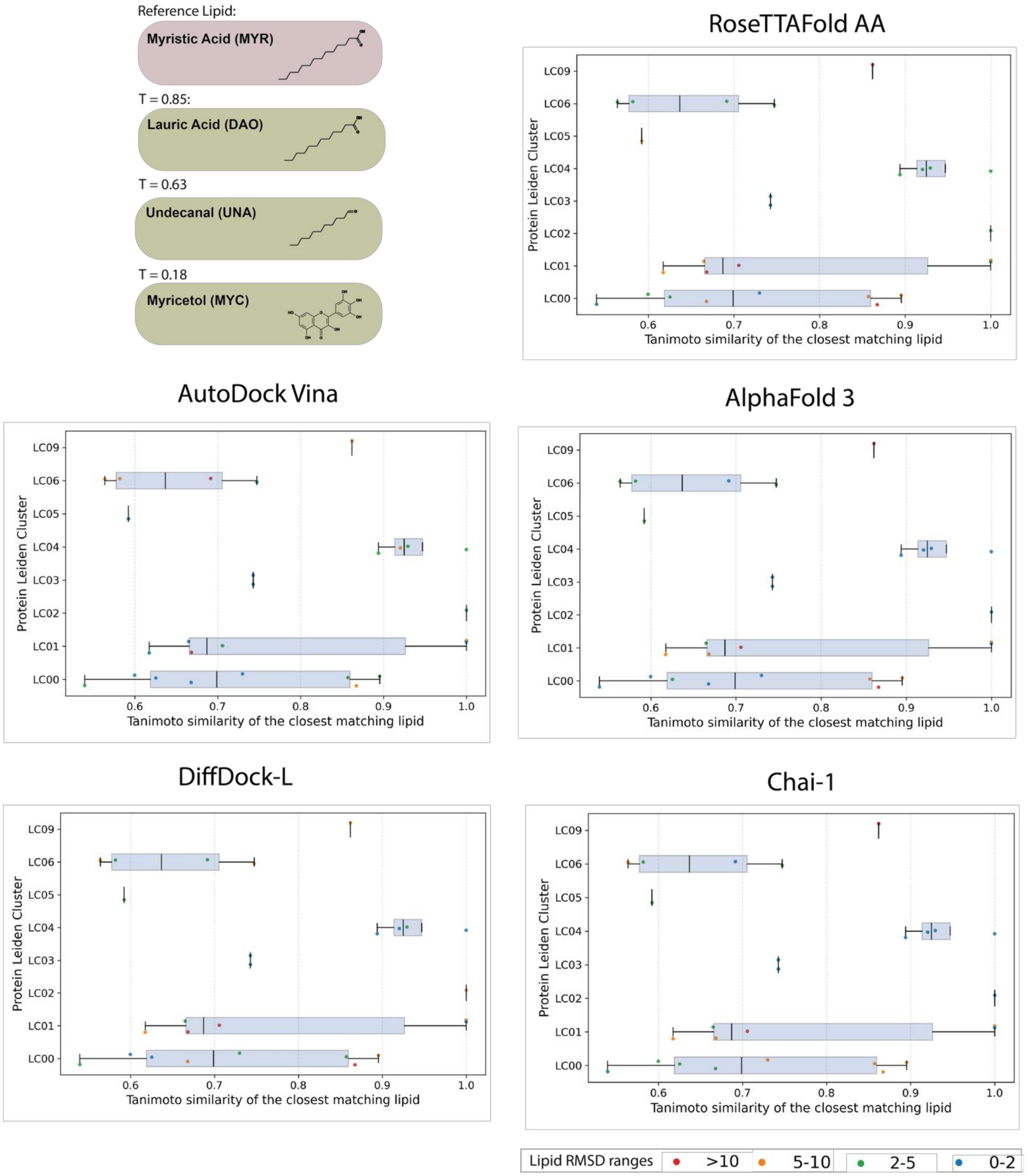

Supplementary Figure 7. *Left*: Example comparison of chemical structures between a reference lipid (myristic acid) and similar/unsimilar lipids (lauric acid, undecanal, and myricetol). *Right:* Box plot showing the distribution of Tanimoto similarity scores for the closest matching lipid in the pre-cutoff dataset for each test set complex, stratified by their protein clusters. Each test set complex is colored according to its lipid all-atom RMSD error from different prediction methods. T denotes the Tanimoto coefficient (similarity score) calculated between the reference lipid and each comparison lipid. Box plots showing the distribution of Tanimoto similarity scores for the closest matching lipid in the

pre-cutoff dataset for each test set complex, stratified by their protein clusters. Each test set complex is colored according to its lipid all-atom RMSD error from different prediction methods. T denotes the Tanimoto coefficient (similarity score) calculated between the reference lipid and each comparison lipid.

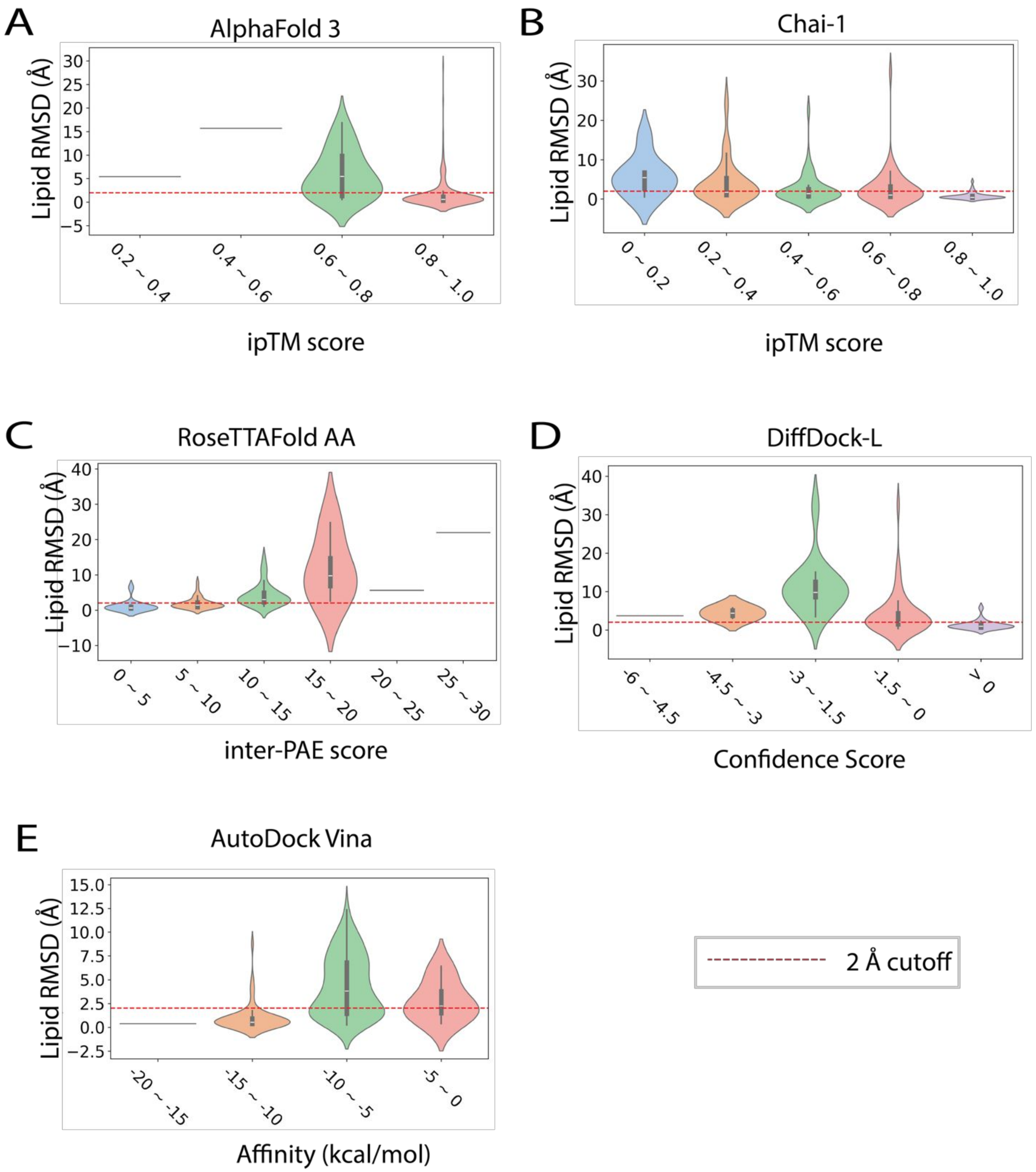

Supplementary Figure 8. Violin plots highlighting the relationship between each method's model discrimination score and the corresponding lipid pose all-atom RMSD

values from the subset of predictions that passed physical validity tests. **A.** ipTM scores of AlphaFold 3-based structure predictions where higher ipTM represents a more confident prediction. **B.** ipTM scores of Chai-1-based structure predictions where higher ipTM represents a more confident prediction. **C.** Inter-PAE scores of RoseTTAFold AA-based structure predictions where lower inter-PAE represents a more confident prediction. **D.** Confidence scores of DiffDock-L docking predictions where a higher confidence score represents a more confident prediction. **E.** Predicted affinity values (kcal/mol) of AutoDock Vina docking predictions where a more negative value presents a more stable binding pose.

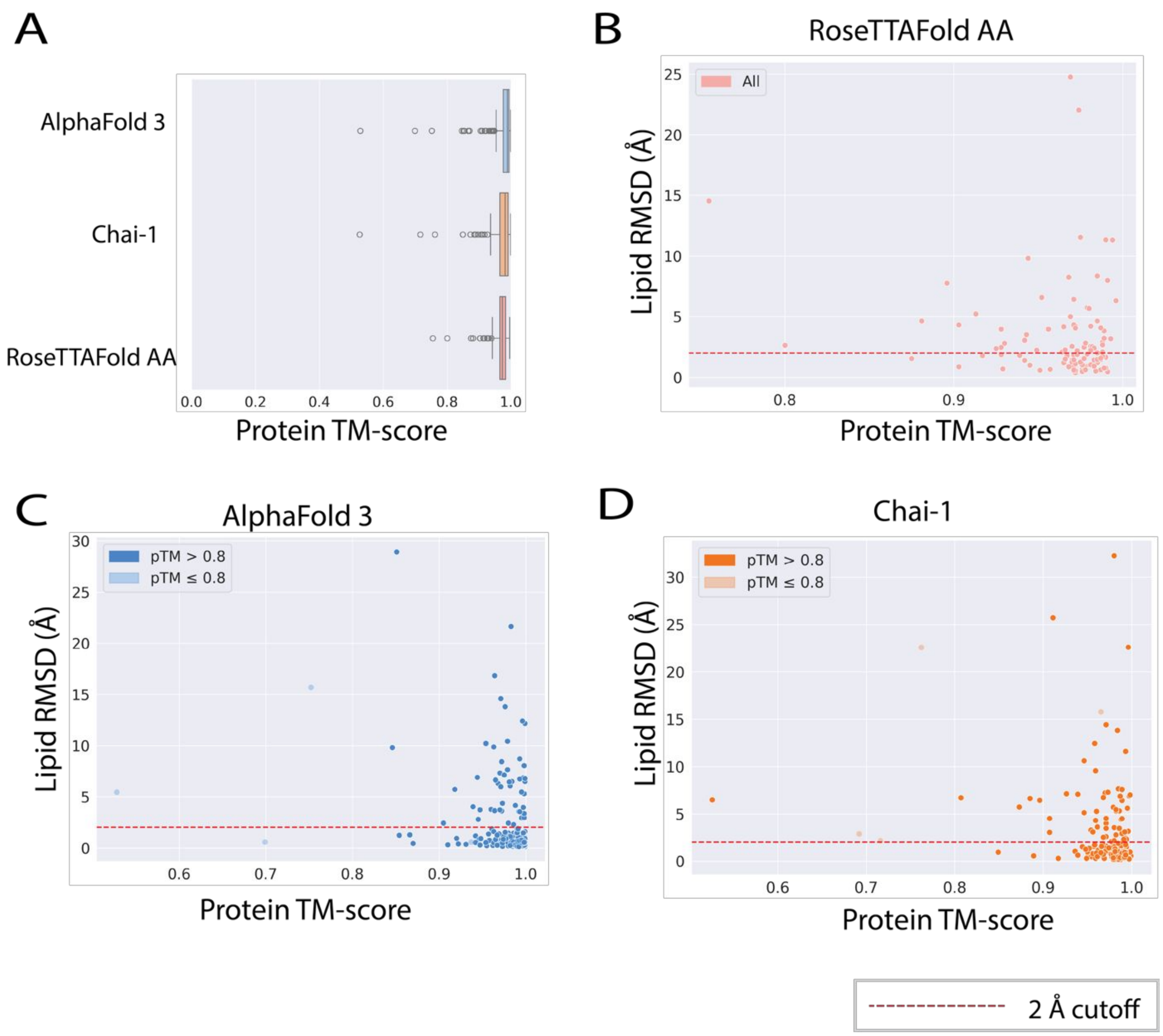

Supplementary Figure 9. Evaluation of protein structure prediction accuracy in lipid–protein complexes from the subset of predictions that passed physical validity tests. TM-scores were calculated between the experimental and predicted protein structures. **A.** Box plot showing the distribution of protein TM-scores predicted by the structure prediction methods evaluated in this study. **B.** to **D.** Scatter plots illustrating the relationship between protein TM-scores and lipid RMSD values. Each point represents a lipid–protein complex in the benchmark dataset. Scatter plots for AlphaFold 3 and Chai-1 are annotated to distinguish predictions with higher confidence (pTM > 0.8) and lower confidence (pTM ≤ 0.8).

**Filtering Step**	**Number of lipid–protein entries**
Original BioDolphin lipid–protein entries	127,359
Structures with no unknown protein residues	127,034
Structures with no modified protein residues	114,728
X-ray structures with resolution less than 2 Å	16,316
Structures with lipid files that can be loaded with RDKit, are complete, and pass the sanitization test	10,703
Structures with no covalent bonds between the lipid and protein	10,491
Structures where the PDB ligand reports do not list clashes	4,828
Structures where the PDB ligand reports do not list stereochemical errors	4,753
Structures where the lipid and protein distance is between 0.2 to 5 Å and no protein symmetry mate exists within 5 Å of the lipid from the source PDB files	2,981
Representative lipid–protein structures based on protein sequence clustering and lipid CCD code were randomly selected to remove redundant pairs	1,075
Manual inspection of non-annular lipid–protein complexes	331

Supplementary Table 1. Filtering workflow and the resulting number of lipid–protein pairs after each step. The full LiPP benchmark set is composed of 331 PDB files of lipid–protein pairs. The filtering workflow was inspired by an analogous workflow used to generate the PoseBusters Benchmark set.
